# Reduced MUNC18-1 Levels, Synaptic Proteome Changes, and Altered Network Activity in *STXBP1*-Related Disorder Patient Neurons

**DOI:** 10.1016/j.bpsgos.2023.05.004

**Published:** 2023-05-30

**Authors:** Annemiek Arienne van Berkel, Hanna Charlotte Andrea Lammertse, Miriam Öttl, Frank Koopmans, Mala Misra-Isrie, Marieke Meijer, Robertino Dilena, Peter Marin van Hasselt, Marc Engelen, Mieke van Haelst, August Benjamin Smit, Sophie van der Sluis, Ruud Franciscus Toonen, Matthijs Verhage

**Affiliations:** a Department of Functional Genomics, Center for Neurogenomics and Cognitive Research, Vrije Universiteit Amsterdam, Amsterdam, the Netherlands; b Functional Genomics, Department of Human Genetics, Center for Neurogenomics and Cognitive Research, Amsterdam Universitair Medische Centra, Amsterdam, the Netherlands; c Department of Molecular and Cellular Neurobiology, Center for Neurogenomics and Cognitive Research, Vrije Universiteit Amsterdam, Amsterdam, the Netherlands; d Department of Human Genetics, Clinical Genetics Section, Amsterdam University Medical Center, Amsterdam, the Netherlands; e Clinical Neurophysiology Unit, Fondazione Istituto di Ricovero e Cura a Carattere Scientifico (IRCCS) Ca’ Granda Ospedale Maggiore Policlinico, Milan, Italy; f Department of Metabolic Diseases, Division of Pediatrics, Wilhelmina Children’s Hospital, University Medical Center Utrecht, Utrecht, the Netherlands; g Department of (Pediatric) Neurology, Amsterdam Universitair Medische Centra, Amsterdam, the Netherlands; h Department of Complex Trait Genetics, Center for Neurogenomics and Cognitive Research, Vrije Universiteit Amsterdam, Amsterdam, the Netherlands; i Department of Child and Adolescence Psychiatry, Section of Complex Trait Genetics, Amsterdam Universitair Medische Centra, Amsterdam, the Netherlands

**Keywords:** iPSC, MUNC18-1, Neurodevelopmental disorder, SNAREopathy, *STXBP1*-related disorder, Synaptic transmission

## Abstract

**Background:**

*STXBP1*-related disorder (*STXBP1*-RD) is a neurodevelopmental disorder caused by pathogenic variants in the *STXBP1* gene. Its gene product MUNC18-1 organizes synaptic vesicle exocytosis and is essential for synaptic transmission. Patients present with developmental delay, intellectual disability, and/or epileptic seizures, with high clinical heterogeneity. To date, the cellular deficits of neurons of patients with *STXBP1*-RD are unknown.

**Methods:**

We combined live-cell imaging, electrophysiology, confocal microscopy, and mass spectrometry proteomics to characterize cellular phenotypes of induced pluripotent stem cell–derived neurons from 6 patients with *STXBP1*-RD, capturing shared features as well as phenotypic diversity among patients.

**Results:**

Neurons from all patients showed normal in vitro development, morphology, and synapse formation, but reduced MUNC18-1 RNA and protein levels. In addition, a proteome-wide screen identified dysregulation of proteins related to synapse function and RNA processes. Neuronal networks showed shared as well as patient-specific phenotypes in activity frequency, network irregularity, and synchronicity, especially when networks were challenged by increasing excitability. No shared effects were observed in synapse physiology of single neurons except for a few patient-specific phenotypes. Similarities between functional and proteome phenotypes suggested 2 patient clusters, not explained by gene variant type.

**Conclusions:**

Together, these data show that decreased MUNC18-1 levels, dysregulation of synaptic proteins, and altered network activity are shared cellular phenotypes of *STXBP1*-RD. The 2 patient clusters suggest distinctive pathobiology among subgroups of patients, providing a plausible explanation for the clinical heterogeneity. This phenotypic spectrum provides a framework for future validation studies and therapy design for *STXBP1*-RD.

Synaptic transmission between neurons requires the tightly controlled SNARE complex machinery to drive synaptic vesicle exocytosis (1,2). Advances in clinical genetics have now linked variants in all 8 core members of the SNARE complex to a clinical spectrum of neurodevelopmental disorders, collectively referred to as SNAREopathies (3). Although variants in different components of the same macromolar protein complex are expected to lead to similar symptoms and disease severity, the clinical phenotype of patients with SNAREopathies is remarkably diverse, not only between genes, but also between patients with variants in the same gene (3, 4, 5, 6, 7). To design rational intervention strategies and personalized treatments, we need a better understanding of the shared and distinctive pathobiology of patients with SNAREopathies.

Variants in the *STXBP1* gene, leading to *STXBP1*-related disorder (*STXBP1*-RD), are most prevalent among the SNAREopathies with an estimated incidence of 1:30,000 (8). *STXBP1* encodes the MUNC18-1 protein, which organizes SNARE complex formation and is essential for synaptic transmission (9,10). All affected patients have neurodevelopmental delay, the majority (80%) experience epileptic seizures, and many experience additional neurological and psychiatric symptoms, yet the type of symptoms and their severity vary substantially (4). *STXBP1* variants range across the whole gene and include missense (50% of cases), nonsense, frameshift, and intronic variants and partial or full deletions. In almost all cases, variants are heterozygous and occur de novo [with few exceptions (11,12)]. To date, no robust correlation between *STXBP1* genotypes and clinical phenotypes has been observed.

The lack of genotype-phenotype correlation, together with in vitro and behavioral animal research, suggests that haploinsufficiency is a plausible underlying disease mechanism. *STXBP1* missense variants cause MUNC18-1 instability, leading to reduced expression levels (13, 14, 15, 16). In addition, several strains of *Stxbp1*^+/−^ mice recapitulate cardinal features of *STXBP1*-RD including seizures and cognitive and other behavioral deficits (13,17,18). In these animal models, synaptic dysfunction has been observed in various brain circuitries, indicating that a reduction in MUNC18-1 levels directly affects synaptic transmission. Moreover, in vitro networks of human embryonic stem cell–derived heterozygous *STXBP1* neurons show deficits in neurotransmitter release and network performance (19,20), and induced pluripotent stem cell (iPSC)–derived neurons from one patient with *STXBP1*-RD showed network activity deficits (21).

However, shared and/or unique pathobiology of patients with *STXBP1*-RD has not yet been established in patients’ own cells. Haploinsufficiency alone is insufficient to explain the substantial clinical diversity, and additional genetic and/or environmental factors probably contribute to this heterogeneity. Current iPSC technology provides the opportunity to study molecular and cellular phenotypes in the patient’s genetic background. By studying a series of patient iPSC-derived neurons (induced neurons), potential pathobiological mechanisms can be identified that are either general to *STXBP1*-RD or distinctive for particular patients or patient subgroups.

The aim of this study was to analyze such shared and distinctive cellular phenotypes in induced neuron lines derived from patients with *STXBP1*-RD. The findings in this discovery study demonstrate reduced MUNC18-1 levels resulting in synaptic proteome dysregulation and altered network activity as underlying pathobiology in neurons derived from patients with *STXBP1*-RD. Interpatient clustering of induced neuron lines, not explained by variant type, was observed at both proteomic and functional level, indicating distinctive pathobiology between patient subgroups.

## Methods and Materials

### Compliance With Ethical Guidelines and Legislation

All experiments were carried out in compliance with relevant laws and institutional guidelines at the Vrije Universiteit in Amsterdam. Details are provided in Supplemental Methods and Materials in Supplement 1.

### Patient Phenotyping

For all patients with *STXBP1*-RD except for the R235Q patient, clinical phenotyping was performed using a questionnaire filled out by the parents/caregivers, and additional or retrospective information was obtained from the medical records. Patients were also assessed by a clinical geneticist and a neurologist for additional clinical assessments.

### Skin Biopsy, iPSC Generation, and Culture

Skin biopsy specimens were performed under sterile conditions after local anesthesia with 2% lidocaine with ephinephrine using a 3-mm skin punch. Fibroblasts were reprogrammed into iPSCs via the CytoTune-iPS 2.0 Sendai Reprogramming Kit (#A16517; Thermo Fisher Scientific Inc.) according to the manufacturer’s instructions. The iPSC cultures were maintained in Essential E8 medium (Thermo Fisher Scientific Inc.) plus 0.1% penicillin-streptomycin on Matrigel-coated plates. Details are provided in Supplemental Methods and Materials in Supplement 1.

The iPSC lines from control individuals were obtained either commercially or from collaborators. Control line 1 was obtained from Coriell Institute for Medical Research (GM25256) and characterized using methods reported in (22) and (23). The donor was 30 years of age at the time of biopsy and is of Asian ancestry. Control line 2 was obtained from Coriell Institute for Medical Research (GM23973) and characterized using methods reported in (24) and (23). The donor was 19 years of age at the time of biopsy and is of White/European ancestry. Control line 3 has been characterized before as reported in (25) and (23) (hVS-88). This line was sampled from a 74-day-old infant. All three control lines were obtained from reprogrammed fibroblasts and derive from male donors.

### Single Nucleotide Polymorphism Analysis and Copy Number Variation Calling

Genomic integrity and identity were assessed using the GSA beadchip GSA MD v1 (Illumina, Inc.) and analysis package iPsychCNV (https://biopsyk.dk/ipsychcnv/). Details are provided in Supplemental Methods and Materials in Supplement 1.

### Neuronal Induction

Neuronal induction of iPSCs was performed via forced expression of the transcription factor NGN2 (Neurogenin-2) and dual SMAD inhibition (26,27). Neurons were plated on a rat glial feeder layer to promote neuronal maturation. Details are provided in Supplemental Methods and Materials in Supplement 1.

### Immunocytochemistry

Neuronal cultures were immunostained as described before (28). Images were acquired on an Eclipse Ti microscope (Nikon Corp.), equipped with confocal scanner model A1R+, using a 40× oil immersion objective (numerical aperture 1.3; Carl Zeiss Microscopy GmbH). Details, including on morphometry on cultured neurons, are provided in Supplemental Methods and Materials in Supplement 1.

### Western Blotting

Western blotting procedures were executed as described before (13). Details are provided in Supplemental Methods and Materials in Supplement 1.

### Quantitative Polymerase Chain Reaction

RNA was isolated using TRIzol and chloroform. Complementary DNA synthesis and quantitative polymerase chain reaction using SYBR Green were performed according to manufacturer’s instructions (Meridian Bioscience). Details are provided in Supplemental Methods and Materials in Supplement 1.

### Mass Spectroscopy

Sample collection, preparation, and mass spectrometry were performed as described previously (29). Gene Ontology (GO) analysis was done in Cytoscape plug-in ClueGO (https://apps.cytoscape.org/apps/cluego). Details are provided in Supplemental Methods and Materials in Supplement 1.

### Electrophysiology

Synapse physiology at single-neuron resolution was recorded by whole-cell patch-clamp electrophysiology of autaptic induced neurons recorded at days in vitro 39 to 42. Analysis was formed in in-house developed MATLAB v2018A (The MathWorks, Inc.) scripts and Clampfit v10.7 (Molecular Devices), further detailed in Supplemental Methods and Materials in Supplement 1.

### Calcium Imaging

Network activity was assessed using Fluo-4, AM (Thermo Fisher Scientific Inc.). Cultures were imaged in normal Tyrode’s solution, and after incubation with 100 μM 4-aminopyridine (MilliporeSigma), analysis was performed in EvA software (30). Details are provided in Supplemental Methods and Materials in Supplement 1.

### Linear Discriminant Analysis

Linear discriminant analysis (LDA) was performed in R/RStudio (v.4.1.1; R Foundation for Statistical Computing; https://www.R-project.org/ and RStudio, Inc.; http://www.rstudio.com/) using the caret and MASS packages. Details are provided in Supplemental Methods and Materials in Supplement 1.

### Statistical Analysis

Statistical analysis was performed using linear mixed-effects models to account for the clustered structure of the data using R/RStudio and lme4 package. Details are provided in Supplemental Methods and Materials and Table S2 in Supplement 1. Sample size numbers are indicated in figure legends, *n/N*: number of observations/number of independent culture batches (i.e., biological replicates). Data were plotted as boxplots with Tukey whiskers that extend to 1.5 times the interquartile range. In plots with overarching comparisons of control individuals and patients, data points outside of this range are plotted as individual dots. In plots comparing groups separately, dots represent individual data points.

## Results

**Figure 1:**
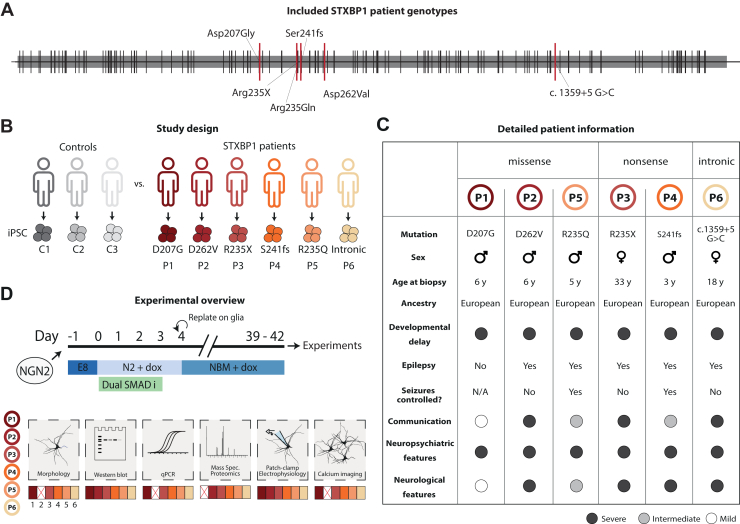
Study design to discover cellular phenotypes in induced neurons derived from patients with *STXBP1*-related disorder (*STXBP1*-RD). **(A)** Overview of *STXBP1*-RD variants. The *STXBP1* gene is depicted; black stripes represent published disease variants, and red stripes show variants included in this study: 2 missense variants D207G and D262V, 2 nonsense variants Arg235X and Lys308X, one frameshift S241fs, and one intronic variant c.1359+5G>C (hereinafter referred to as intronic). Exons and introns are not depicted for clarity. **(B)** Study design. iPSCs were generated from 6 *STXBP1*-RD patients and 3 healthy control individuals. **(C)** Summary table of the clinical phenotypes of the included *STXBP1*-RD patients. Severity of clinical symptoms is indicated by shading (black = severe; gray = intermediate; white = mild). Detailed information is provided in Table S1 in Supplement 1. **(D)** Schematic presentation of the experimental design. iPSCs were induced to glutamatergic induced neurons via forced expression of NGN2. After 39–42 days in culture, induced neurons were examined by confocal microscopy, liquid chromatography–tandem mass spectrometry proteomics, patch clamp electrophysiology, and calcium imaging. Sporadically, technical failures prevented analyses of individual patient lines, as indicated by a red X (see main text). For each parameter measured in this study, overarching effects between control individuals and patients were assessed by comparing pooled measurements from all control and all patient lines, while accounting for the multilevel (nested) data structure (see Methods and Materials). In addition, patient-specific differences were assessed by comparing measurements from each patient line separately with the pooled measurements from the control lines, again accounting for the multilevel structure. C, control; iPSC, induced pluripotent stem cell; N/A, not applicable; P, patient; qPCR, quantitative polymerase chain reaction; Spec., spectrometry.

We generated a cohort of iPSC lines from 6 patients with *STXBP1*-RD, each carrying a different *STXBP1* de novo variant (Figure 1A). One variant introduced a stop codon (R235X); one introduced a frameshift mutation (S241fs); one predicted introduction of a splice site (c.1359+5 G>C; intronic); and 3 were missense variants (D207G, D262V, R235Q), of which the former may create a (cryptic) splice site according to SpliceFinder (31). The 6 patient-derived lines were compared with 3 independent iPSC lines from unrelated, healthy individuals (control lines 1–3) (Figure 1B). The occurrence and severity of clinical symptoms varied substantially between patients (Figure 1C; Table S1 in Supplement 1), which is typical for the *STXBP1*-RD patient population (4). Induced neurons were cultured on rodent astrocytes to promote neuronal maturation for 39 to 42 days in vitro, at which time point the induced neurons are synaptically mature [see below and (28)]. Six independent inductions were performed for all 9 experimental groups (6 patient-derived lines and 3 control lines) and subjected to an array of functional assays (Figure 1D): neuronal morphology and synaptogenesis, protein levels (using Western blotting and immunocytochemistry), messenger RNA (mRNA) levels, proteomics, synapse physiology (patch clamp), and network activity (using calcium imaging). Sporadically, some inductions for specific experimental groups were not included in these analyses due to technical failures or biological issues, leading to the exclusion of at most one patient line in 3 of the 6 functional assays (Figure 1D; see Tables S1 and S2 in Supplement 1 for details on group sizes and statistical tests).

### iPSC-Derived Neurons From 6 *STXBP1*-RD Patients Have Normal Morphology and Synapse Numbers

**Figure 2:**
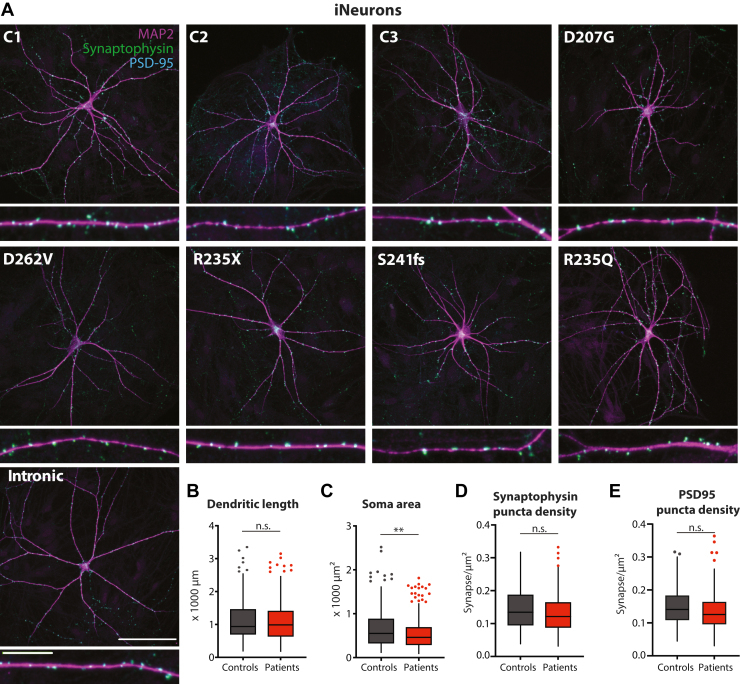
Neuronal morphology and synapse formation are normal in patient-derived induced neurons. **(A)** Typical examples of single induced neurons from each control and *STXBP1*-related disorder (*STXBP1*-RD) line stained for dendritic marker (MAP2), presynapse marker (synaptophysin) and postsynaptic PSD-95. Scale bar = 100 μm; scale bar zoom = 20 μm. **(B)** Dendritic length was not different between control lines and *STXBP1*-RD induced neuron lines. *n/N* = 57–96/3–6. **(C)** Soma area was significantly smaller for *STXBP1*-RD induced neurons at group level. *n/N* = 57–96/3–6. **(D)** Density of presynaptic puncta, labeled by synaptophysin immunostaining, was not different between control and *STXBP1*-RD induced neuron lines. *n/N* = 53–99/3–6. **(E)** Density of postsynaptic puncta, labeled by PSD-95 immunostaining, was not different between control and *STXBP1*-RD induced neuron lines. *n/N* = 53–99/3–6. Data in panels **(B–E)** are presented in Tukey plots, where data outside of 1.5 times the interquartile range are plotted individually. Statistical details are listed in Table S2 in Supplement 1. ∗∗*p* < .01. *n/N*: number of neurons/number of independent replicates. C, control; n.s., not significant.

To assess whether *STXBP1* variants affect neuronal morphology and synapse formation/maintenance, single induced neurons were immunostained for dendritic marker MAP2, presynaptic protein synaptophysin, and postsynaptic protein PSD-95 (Figure 2A). Neuronal morphology, dendritic complexity, and synapse density were comparable between *STXBP1*-RD and control induced neurons except for a small reduction in soma size (Figure 2B–E; Figure S1A–C in Supplement 1). Thus, neuronal morphology and synapse density are not affected in *STXBP1*-RD induced neurons.

### MUNC18-1 Protein and RNA Levels Are Reduced in *STXBP1*-RD Neurons

**Figure 3:**
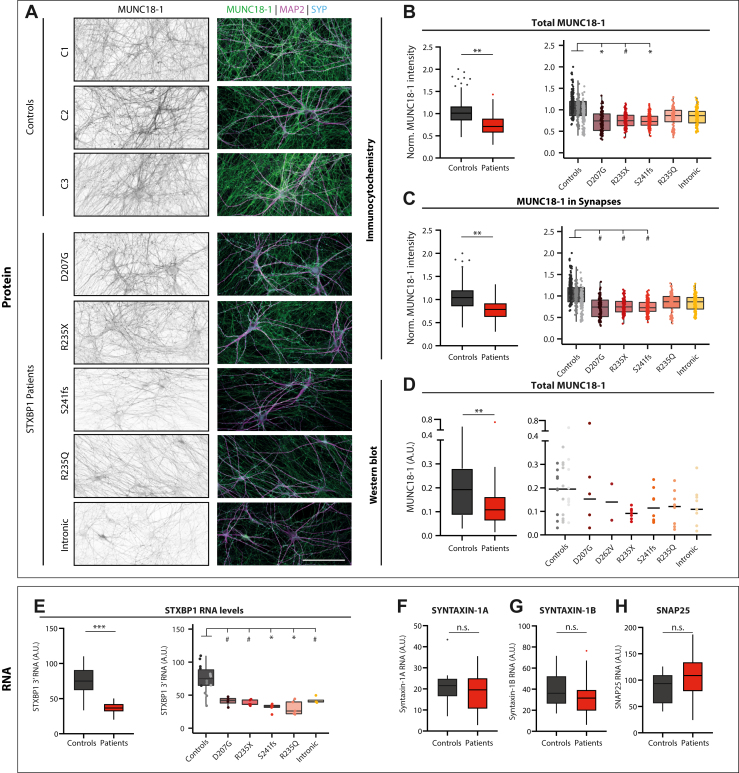
MUNC18-1 protein and RNA levels are reduced in *STXBP1*-related disorder (*STXBP1*-RD) induced neurons. **(A)** Typical examples of induced neurons stained for MAP2 (dendritic marker), synaptophysin (synaptic marker), and MUNC18-1. Scale bar = 100 μm. **(B)** (Left panel) Neuronal MUNC18-1 levels were reduced in *STXBP1*-RD induced neurons compared with control induced neurons. (Right panel) Significant reductions of MUNC18-1 levels were observed for D207G and S241fs induced neurons. All intensities were acquired with the same detection settings. Afterward average intensities were normalized to the average of the 3 control lines. **(C)** (Left panel) *STXBP1*-RD induced neurons showed reduced levels of synaptic MUNC18-1. (Right panel) At the individual patient level, no significant effects were observed. **(D)** MUNC18-1 levels were quantified by immunoblotting and normalized to the levels of gamma-tubulin. *STXBP1*-RD cultures showed reduced total MUNC18-1 levels. **(E)** (Left panel) *STXBP1* RNA levels (using *STXBP1* 3′ primers) were lower in *STXBP1*-RD induced neurons compared with control induced neurons. (Right panel) Significantly reduced levels were observed for S241fs and R235Q. **(F)** Syntaxin-1A RNA levels were not different in the group-level comparison between *STXBP1*-RD and control induced neurons. **(G)***STXBP1*-RD induced neurons did not show lower syntaxin-1B RNA levels. **(H)** Group-level SNAP25 RNA levels were not different in *STXBP1*-RD induced neurons. Data on the left of panels **(B–E)** and all of panel **(F)** are presented in Tukey plots, where data outside of 1.5 times the interquartile range are plotted individually. Data on the right of panels **(B)** and **(C)** are presented in Tukey plots with dots representing individual neurons; in panels **(D)** and **(E)** individual data points are shown. Immunocytochemistry *n/N* = 98–130/5; Western blot *N*: 2–10; quantitative polymerase chain reaction *N*: 4–5. ∗*p* < .05, ∗∗*p* < .01, ∗∗∗*p* < .001, #*p* < .1. Statistical details are listed in Table S2 in Supplement 1. *n/N*: number of neurons/number of independent replicates. A.U., arbitrary unit; C, control; Norm., normal; n.s., not significant; SYP, synaptophysin.

Next, we investigated whether MUNC18-1 protein and RNA levels were affected in induced neurons from patients with *STXBP1*-RD. MUNC18-1 protein levels, measured using semiquantitative immunocytochemistry with an antibody that detects both known splice variants (Figure 3A), were significantly reduced in *STXBP1*-RD induced neurons in total, in MAP2-positive dendrites, and in synaptophysin-1–positive synapses (Figure 3B, C; Figure S2A, B in Supplement 1). MUNC18-1 immunocytochemistry did not reveal abnormal protein accumulation or aggregation, as observed in overexpression studies and transgenic nematodes (14), or other abnormal MUNC18-1 distribution. Western blot analysis of cell lysates confirmed the reduction in MUNC18-1 levels (Figure 3D). To examine RNA levels, quantitative polymerase chain reaction was performed on cell lysates with primers that detect both known splice variants of MUNC18-1 (Figure S2C in Supplement 1). All MUNC18-1 transcript levels were significantly reduced in *STXBP1*-RD induced neurons, including the neurons expressing missense variant D207G, which according to SpliceFinder (31) may have a (cryptic) splice site (Figure 3E; Figure S2D, E in Supplement 1). Because null mutation of *Stxbp1* in mouse neurons leads to changes in mRNA levels of syntaxin-1A and SNAP25 (32), we also assessed these 2 transcripts. However, no shared patient effects were found for syntaxin-1A, syntaxin-1B, or SNAP25 mRNA levels (Figure 3F–H; Figure S2F–H in Supplement 1). Hence, MUNC18-1 protein and mRNA levels are reduced in *STXBP1*-RD induced neurons.

### Expression of Synaptic and RNA Processing Proteins Is Altered in *STXBP1*-RD Neurons

**Figure 4:**
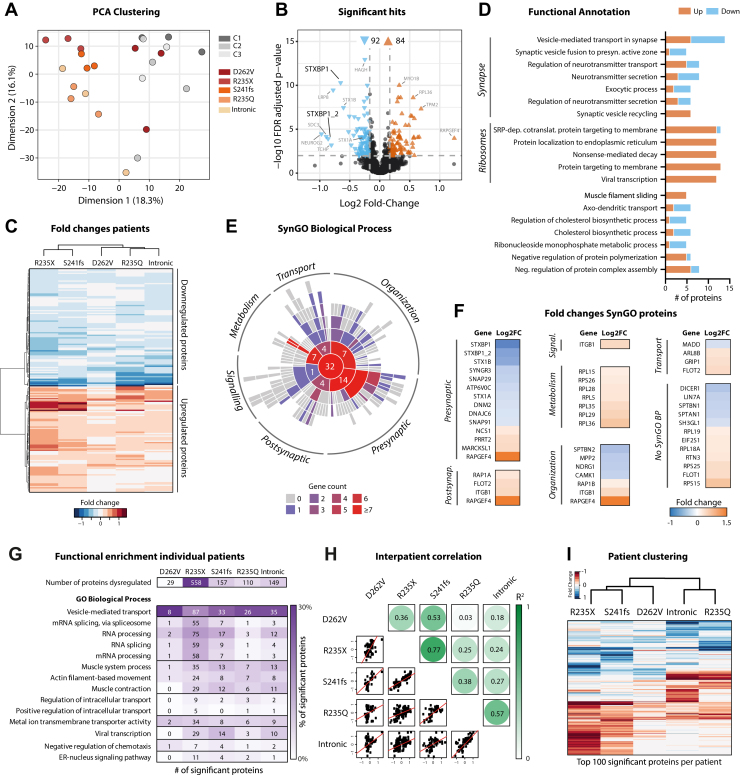
Proteins related to synaptic and RNA biological processes are most severely affected in *STXBP1*-related disorder (*STXBP1*-RD) induced neurons. **(A)** PCA of peptide abundance levels performed on all detected proteins showing PC1 (18.3% of variance explained) and PC2 (16.1% of variance explained) of *STXBP1*-RD and control induced neurons. *N* = 3 independent replicates. **(B)** Volcano plot showing 176 (92 downregulated, 84 upregulated) proteins significantly regulated in *STXBP1*-RD induced neurons compared with control lines. *STXBP1*/MUNC18-1 was one among the top regulated proteins. **(C)** Heatmap visualizing patient-specific log_2_-FC of the 176 significant proteins. Directionality and effect sizes of regulated proteins were comparable across patients. Fold changes were capped at −1.5 and 1.5 for visualization. Hierarchical clustering was used to visualize patient subgroups based on similarity in log_2_-FC. **(D)** Functional annotation of the significant hits (minimum 5 proteins per term, minimum 5% of the GO term) covered 58 proteins. The number of proteins associated with every GO term are shown. GO terms related to the synapse and RNA processes were most prominent. **(E)** Of the significant proteins, 44 were annotated in SynGO, of which 32 were categorized in SynGO biological processes. A sunburst plot with color-coded gene counts of every GO term (including child terms) is shown. **(F)** Group-level log_2_-FC for the 44 SynGO proteins. **(G)** Top row shows the number of significantly regulated proteins in 5 *STXBP1*-RD induced neuron lines. Functional GO enrichment of patient-level contrasts is shown below. The number of proteins associated with significantly enriched GO biological processes is depicted for every individual line, color coded by the percentage of all regulated proteins per line. **(H)** Proteomic similarity matrix. Coefficients of determination (*R*^2^) were calculated between every *STXBP1*-RD pair, based on significantly regulated proteins in at least one of the 2 lines. The upper part of graph shows *R*^2^ values, and lower part shows scatterplots of the log_2_-FC of the included proteins. Red line indicates a robust regression fit. **(I)** Heatmap visualizing log_2_-FC of proteins that were in the top 100 significant proteins in at least one of the *STXBP1*-RD induced neuron lines. Fold changes were capped at −1 and 1 for visualization. Hierarchical clustering was used to visualize patient subgroups based on similarity in log_2_-FC. C, control; cotranslat., cotranslation; ER, endoplasmic reticulum; FC, fold change; FDR, false discovery rate; GO, Gene Ontology; mRNA, messenger RNA; Neg, negative; PCA, principal component analysis; presyn, presynaptic; SRP-dep, signal recognition particle–dependent.

To assess proteome alterations in *STXBP1*-RD induced neurons, quantitative mass spectrometry was performed on induced neuron cultures. A mean (SD) of 3798 (52.8) proteins were detected per sample. Principal component (PC) analysis of peptide abundance levels showed that the 2 main components (PC1: 18.3%; PC2: 16.1%) separated *STXBP1*-RD cultures from control lines, with the exception of D262V induced neurons, which clustered with control lines (Figure 4A). Differential expression analysis identified 176 proteins significantly dysregulated in *STXBP1*-RD cultures (92 down and 84 up) (Figure 4B; see Table S4 in Supplement 2 for details). *STXBP1*/MUNC18-1 was among the most downregulated proteins. Directionality and fold changes of the 176 dysregulated proteins were highly comparable across induced neuron lines (Figure 4C). Functional annotation with GO terms showed that biological processes related to synapse function and RNA processes, including nonsense-mediated decay, were most affected in *STXBP1*-RD induced neurons (Figure 4D). To specifically examine dysregulation of synaptic proteins, significant hits were analyzed in the SynGO knowledge base (33). Of the 176 regulated proteins, 44 (26%) were annotated in SynGO, of which 32 were mapped to biological processes in the synapse. Dysregulated proteins were found in all major synapse categories (Figure 4E). Equal proportions were either downregulated or upregulated, although this balance was shifted in different subcategories: the majority of presynaptic proteins were downregulated, whereas differentially expressed postsynaptic proteins were largely upregulated (Figure 4F). In sum, *STXBP1*-RD induced neurons showed substantial changes in protein expression, especially for proteins related to synaptic function and RNA metabolism.

### Distinctive Proteome Regulation Among Interpatient Clusters

We next tested how the altered proteomes of the individual *STXBP1*-RD induced neuron lines were interrelated. Affected biological processes were comparable across patient lines except for D262V induced neurons and largely overlapped with GO terms at group level (Figure 4G; compare to Figure 4D). The highest numbers of differentially expressed proteins were found for the GO term vesicle-mediated transport. Relative numbers of proteins within biological processes, scaled to the total number of dysregulated proteins, were generally comparable between induced neuron lines (color coding in Figure 4G). Hence, these interindividual comparisons revealed both unique and shared proteomic alterations, with strong overall similarities in vesicle transport and RNA-processing protein expression.

To assess the extent of similarity in proteomic changes between *STXBP1*-RD induced neuron lines, we calculated the coefficient of determination (*R*^2^) between all *STXBP1*-RD pairs (Figure 4H). Large similarities were observed in 2 patient clusters: R235X-S241fs-D262V (cluster A) and R235Q-intronic (cluster B) induced neurons. This interpatient clustering was further demonstrated by hierarchical clustering of the fold changes of the top 100 dysregulated proteins per induced neuron line (Figure 4I). Thus, increased proteomic similarity was observed for R235X-S241fs-D262V induced neurons as well as for R235Q-intronic induced neurons.

### *STXBP1*-RD Neurons Maintain Normal Synaptic Transmission at Single Cell Level

**Figure 5:**
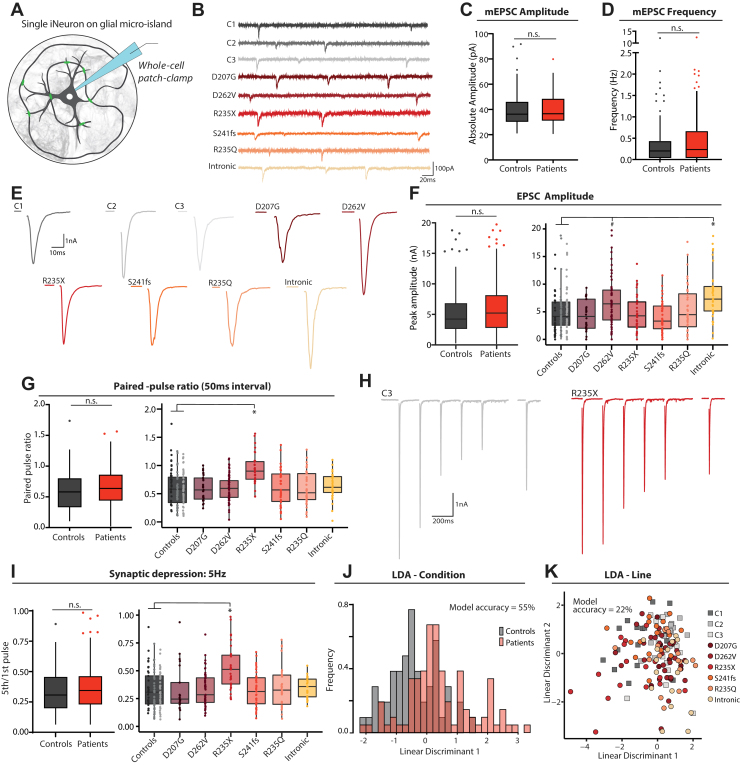
No overarching synaptic changes in single *STXBP1*-related disorder (*STXBP1*-RD) induced neurons. **(A)** Single induced neurons are grown on micro-islands of pregrown glial cells so that the induced neuron forms synapses onto itself. Thus, a single patch pipette can be used to simultaneously stimulate presynaptically and record the postsynaptic response. **(B)** Typical example traces of spontaneous synaptic activity in control and *STXBP1*-RD induced neurons. **(C)** No group-level differences were observed in mEPSC amplitude (for graphs showing the data per patient line, see Figure S5C in Supplement 1). *n/N* = 10–52/3–6. **(D)** No group-level differences were observed in mEPSC frequency (for graphs showing the data per patient line, see Figure S5B in Supplement 1). *n/N* = 8–35/3–6. **(E)** Typical traces of evoked EPSCs of *STXBP1*-RD and control induced neuron lines. **(F)** (Left panel) EPSC amplitude was not different in *STXBP1*-RD induced neurons at the group level. (Right panel) At the patient-specific level, intronic induced neurons had a significantly higher EPSC amplitude. *n/N* = 26–61/3–6. **(G)** (Left panel) No group-level differences were observed in paired-pulse ratio. (Right panel) R235X induced neurons showed a significantly higher paired-pulse ratio compared with control induced neurons. *n/N* = 27–57/3–6. **(H)** Typical traces of a stimulation paradigm of 5 action potentials at 5 Hz, followed by a single pulse 2 seconds following the end of the train. Examples for control line C3 (gray) and patient line R235X (red) are shown. **(I)** (Left panel) No group-level differences were observed between patient and control lines in synaptic depression in response to a 5 Hz train. However, induced neurons with R235X variant showed a significantly higher synaptic depression ratio compared with control neurons. *n/N* = 21–55/3–6. **(J)** Linear discriminant analysis using all electrophysiological parameters had an accuracy of 55% [n.s. according to (40)] to discriminate between *STXBP1*-RD and control induced neurons. **(K)** Linear discriminant analysis using all electrophysiological parameters had an accuracy of 22% [n.s. according to (40)] to predict line identity. Linear discriminants 1 and 2 are shown. Data in panels **(C)** and **(D)** and the left of panels **(F)**, **(G)**, and **(I)** are presented in Tukey plots, where data outside of 1.5 times the interquartile range are plotted individually. Data on the right of panels **(F)**, **(G)**, and **(I)** are presented in Tukey plots with dots representing individual neurons. ∗*p* < .05, #*p* < .1. Statistical details are listed in Table S2 in Supplement 1. *n/N*: number of neurons/number of independent replicates. C, control; LDA, linear discriminant analysis; mEPSC, miniature excitatory postsynaptic current; n.s., not significant.

Given the known essential role of MUNC18-1 in synaptic transmission and the reduced MUNC18-1 levels in *STXBP1*-RD induced neurons, we studied synaptic transmission in these neurons. *STXBP1*-RD neurons and control lines were cultured as single neurons on glial micro-islands, and measured using patch clamp electrophysiology (Figure 5A). This culture system has previously been pivotal in unraveling the molecular and cellular roles of MUNC18-1 in murine neurons (34, 35, 36, 37, 38, 39). No differences were observed in spontaneous synaptic currents (amplitude in Figure 5B,C and Figure S4A in Supplement 1; frequency in Figure 5D and Figure S4B in Supplement 1). In response to action potential stimulation, no overarching differences were observed except for a larger excitatory postsynaptic current amplitude for intronic induced neurons (Figure 5E, F); no difference was observed in excitatory postsynaptic current charge (Figure S4C in Supplement 1). Short-term plasticity was assessed by a paired-pulse stimulation with a 50-ms interval and 5, 10, and 20 Hz train stimulations (Figure 5G–I; Figure S5A–F in Supplement 1). No overarching differences between control and *STXBP1*-RD induced neurons were found, but R235X induced neurons had a higher paired-pulse ratio (Figure 5H) and reduced synaptic depression during train stimulation (Figure 5I; Figure S5B, C in Supplement 1). No group-level differences were observed in recovery after synaptic depression or after depletion of the total release-ready synaptic vesicle pool (Figure S5 in Supplement 1). The size of the total pool of readily releasable vesicles was not different for any patient line except for intronic induced neurons (Figure S5H, I in Supplement 1). In sum, no difference in synapse physiology was observed between control and *STXBP1*-RD neurons except for a few patient-specific effects.

LDA was applied to investigate whether the combined set of synapse physiology parameters discriminates *STXBP1*-RD single neurons from control neurons (Figure 5J). The model accuracy was 55%, which is not significantly different from chance level (40). Subsequently, LDA was performed on all separate lines (Figure 5K), yielding a model accuracy of 22%, again not significant from chance level. Plotting linear discriminants 1 and 2 revealed no clustering of control and *STXBP1*-RD lines. Hence, LDA confirms that single-cell synapse physiology parameters are similar between control and *STXBP1*-RD patient lines.

### Altered Activity and Reduced Synchronicity in *STXBP1*-RD Neuron Networks

**Figure 6:**
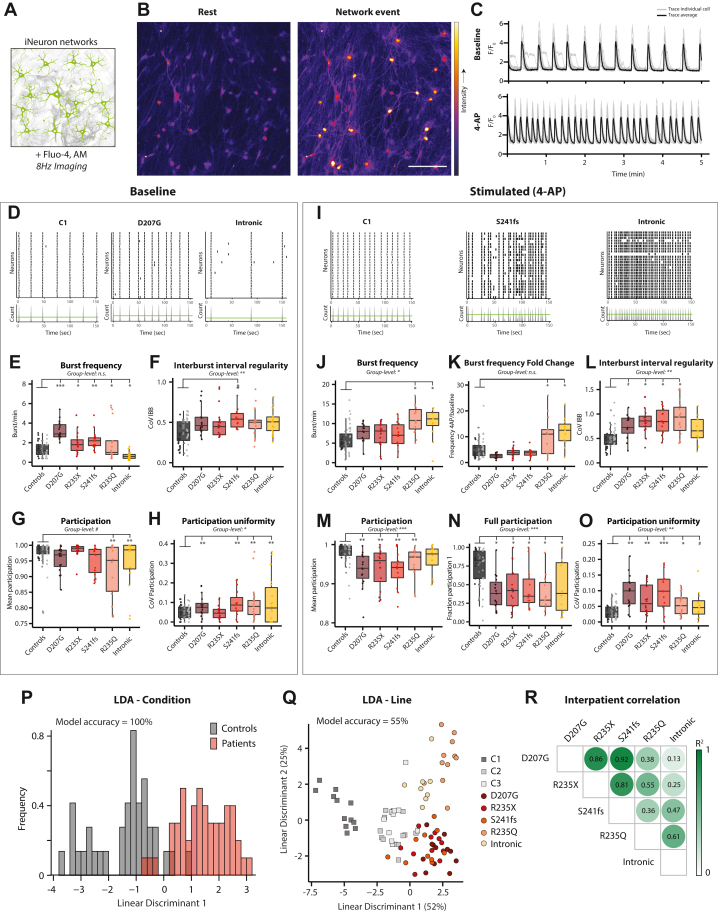
Altered burst activity and reduced synchronicity in networks of *STXBP1*-related disorder (*STXBP1*-RD) induced neurons. **(A)** Induced neuron networks were incubated with Fluo-4 AM and imaged at 8 Hz for 5 minutes at baseline, and 5 minutes after 4-AP administration to study network activity dynamics. **(B)** Typical example of induced neuron network loaded with Fluo-4 AM dye. Fluorescence increases on calcium influx. Scale bar = 100 μm. **(C)** Activity matrix of induced neuron network at baseline and after 4-AP incubation. Fluorescent signal traces of single induced neurons (gray) and average (black) over time (x-axis) are shown. Highly synchronous activity between induced neurons was observed. 4-AP increases event frequency. **(D)** Typical examples of control line C1, patient line D207G, and intronic induced neuron networks at baseline conditions. (Top panel) Black dots represent the start of events in single neurons (y-axis) over time (x-axis). (Bottom panel) Summation of events in individual neurons (y-axis) across one induced neuron network over time (x-axis). Peaks above the green threshold represent synchronous network events. **(E)** Burst frequency was not different at the group level. In patient-level comparisons, an increase in baseline burst frequency was observed for D207G, R235X, S241fs, and R235Q induced neuron networks compared with control networks. Intronic networks showed significantly reduced burst frequency (for group-level graph, see Figure S7A in Supplement 1). **(F)** CoV of interburst intervals was significantly higher in *STXBP1*-RD induced neuron networks. In patient-level comparisons, no significant effects were found (for group-level graph, see Figure S7C in Supplement 1). **(G)** No significant difference was found for mean participation between *STXBP1*-RD and control networks in group-level comparison. Mean participation at baseline was significantly reduced in R235Q and intronic induced neurons (for group-level graph, see Figure S8A in Supplement 1). **(H)** CoV of participation at baseline was significantly increased in *STXBP1*-RD induced neuron networks, which was also significant in D207G, S241fs, R235Q, and intronic induced neuron networks in patient-level comparisons (for group-level graph, see Figure S8C in Supplement 1). **(I)** Typical examples of control line C1, patient line S241fs, and intronic induced neuron networks after 4-AP application. (Top panel) Black dots represent the start of events in single neurons (y-axis) over time (x-axis). (Bottom panel) Summation of events in individual neurons (y-axis) across one induced neuron network over time (x-axis). Peaks above the green threshold represent synchronous network events. **(J)** Group-level comparison revealed increased burst frequency for *STXBP1*-RD induced neuron networks. In patient-level comparisons, R235Q and intronic induced neuron networks showed increased burst frequency compared with control induced neuron networks in 4-AP conditions (for group-level graph, see Figure S7I in Supplement 1). **(K)** Fold change of burst frequency from baseline to 4-AP was not different in group-level comparison. Patient-level comparison showed significant increases for R235Q and intronic induced neuron networks compared with control networks. **(L)** CoV of interburst intervals in 4-AP conditions was significantly increased in *STXBP1*-RD induced neuron networks. In patient-level comparisons, significance was reached for R235X, S241fs, and R235Q induced neuron networks (for group-level graph, see Figure S7L in Supplement 1). **(M)** Mean participation was reduced in *STXBP1*-RD induced neuron networks. In patient-level comparisons, D207G, R235X, S241fs, and R235Q networks showed significantly reduced mean participation (for group-level graph, see Figure S8E in Supplement 1). **(N)** The fraction of neurons participating in all network events (participation index = 1) was significantly lower in *STXBP1*-RD induced neuron networks (for patient-level graph, see Figure S8F in Supplement 1). **(O)** CoV of participation in 4-AP was significantly increased in D207G, R235X, S241fs, R235Q, and intronic induced neuron networks. **(P)** Linear discriminant analysis using all network parameters (including all in Figures S7 and S8 in Supplement 1) had an accuracy of 100% to discriminate between *STXBP1*-RD and control induced neurons. **(Q)** Linear discriminant analysis using all network parameters (including all in Figures S7 and S8 in Supplement 1) had an accuracy of 55% to predict line identity. Shown are linear discriminants 1 and 2. **(R)** Coefficients of determination (*R*^2^) were calculated between every *STXBP1*-RD pair, based on all network parameters. *R*^2^ values ranged between 0.07 and 0.86. Data in panels **(E–H)** and **(J–O)** are presented in Tukey plots with dots representing individual networks. Group-level significance (i.e., overarching effects between control and patient groups) are indicated below graph title. Baseline *n/N* = 16–19/5. 4-AP *n/N* = 12–18/5. ∗*p* < .05, ∗∗*p* < .01, ∗∗∗*p* < .001, #*p* < .1. Statistical details are listed in Table S2 in Supplement 1. *n/N*: number of neurons/number of independent replicates. 4-AP, 4-aminopyridine; C, control; CoV, coefficient of variation; LDA, linear discriminant analysis; n.s., not significant.

Functional deficits caused by *STXBP1* variants may not be revealed at the single cell level, but rather become apparent in neuronal networks. To test this, we examined activity and dynamics of *STXBP1*-RD induced neuron networks (consisting of excitatory neurons only) using fluorometric calcium imaging (Figure 6A, B). After 40 days in culture, control induced neuron networks showed rhythmic and highly synchronous burst events lasting for several seconds (Figure 6C), as shown previously (41,42).

Compared with control networks, *STXBP1*-RD induced neuron networks showed diverging effects on burst frequency: increased burst frequency for D207G, R235X, S241fs, and R235Q induced neuron networks, whereas intronic induced neuron networks showed reduced burst frequency (Figure 6D, E; Figure S6A in Supplement 1). Consequently, the average interburst interval was reduced for D207G, R235X and S241fs induced neuron networks and increased for intronic networks (Figure S6B in Supplement 1). Regularity of burst activity was assessed by the coefficient of variation (CoV) of interburst intervals. CoV values were higher in all *STXBP1*-RD induced neuron networks compared with control networks (Figure 6F; Figure S6C in Supplement 1), indicating increased irregularity of burst activity. In addition, several burst event characteristics were affected in *STXBP1*-RD induced neuron networks, including a significantly smaller event area, shorter event duration, and shorter rise time, whereas no effects were observed for mean event amplitude (Figure S6D–H in Supplement 1). In control induced neuron networks, nearly all induced neurons participated in a burst event, resulting in a mean participation index close to 1 (Figure 6G). In contrast, R235Q and intronic induced neuron networks showed significantly reduced participation (Figure 6G; Figure S7A in Supplement 1), and the fraction of neurons participating in all burst events was lower in D207G and R235Q induced neuron networks (Figure S7B in Supplement 1). The CoV of the participation index (a measure of participation uniformity) was increased in D207G, S241fs, R235Q, and intronic *STXBP1*-RD induced neuron networks (Figure 6H; Figure S7C in Supplement 1). Taken together, baseline activity frequency, burst characteristics, and network synchronicity of *STXBP1*-RD induced neuron networks are altered compared with control networks with additional, patient-specific effects in burst frequency and synchronicity.

To assess phenotypes of challenged induced neuron networks, cultures were incubated with the potassium channel blocker 4-aminopyridine (4-AP). In control induced neuron networks, 4-AP caused a 5-fold increase in burst frequency (Figure 6I, J; Figure S6J in Supplement 1). Compared with control networks, R235Q and intronic induced neuron networks showed increased burst activity (Figure 6I, J; Figure S6I in Supplement 1). Strikingly, both of these patient induced neuron networks showed a relatively low burst frequency during rest (Figure 6E), and therefore the increased frequency after 4-AP indicates that these 2 induced neuron networks had a larger fold change than the others (i.e., 4-AP–induced frequency jump) (Figure 6K). Interburst interval values were reduced in *STXBP1*-RD induced neuron networks, again most prominently in R235Q and intronic networks (Figure S6J in Supplement 1). Interburst interval CoV (burst activity irregularity) upon 4-AP was increased in *STXBP1*-RD induced neuron networks (Figure 6L; Figure S6K in Supplement 1). Similar to baseline conditions, event area, event duration, rise time, and decay time all were reduced in *STXBP1*-RD induced neuron networks, while the mean event amplitude was not different (Figure S6L–P in Supplement 1). Upon 4-AP, phenotypes in synchronicity parameters were exacerbated compared with baseline. The mean participation and the fraction of neurons participating in all events were strongly reduced in *STXBP1*-RD induced neuron networks (Figure 6M, N; Figure S7D, E in Supplement 1). Participation CoV values were significantly increased in all *STXBP1*-RD induced neuron networks, yet most prominently for D207G, R235X, and S241fs networks (Figure 6O; Figure S7F in Supplement 1). Taken together, 4-AP unmasked stronger differences between control and patient induced neuron networks in burst frequency, irregularity, and synchronicity and identified 2 subgroups, R235Q and intronic induced neuron networks, characterized by a 4-AP–induced frequency jump, and D207G, R235X, and S241fs networks, characterized by strong reduced participation uniformity.

To assess whether network activity parameters discriminate *STXBP1*-RD induced neuron networks from control networks, LDA was first performed on all network parameters. LDA reached an accuracy of 100% when discriminating based on condition (Figure 6P; Figure S8A in Supplement 1). Second, LDA performed on line identity correctly classified 55% of the networks, also significant from chance level (Figure 6Q). Linear discriminant 1 (accounting for 52% of model) discriminated control induced neuron networks from *STXBP1*-RD networks (Figure 6Q). Linear discriminant 2 (accounting for 25% of model) then separated the 4-AP–induced frequency jump subgroup (R235Q and intronic induced neuron networks) from the participation uniformity subgroup (D207G, R235X, and S241fs induced neuron networks). To further explore the degree of interpatient similarities, *R*^2^ values between *STXBP1*-RD induced neuron networks were determined (Figure 6R). High *R*^2^ values were observed for the 4-AP–induced frequency jump subgroup and the participation uniformity subgroup. In sum, differences in network activity parameters are effective to discriminate *STXBP1*-RD and control induced neuron networks. Among *STXBP1*-RD induced neuron networks, interpatient clustering was observed between the participation uniformity subgroup (D207G, R235X, and S241fs) and the frequency jump subgroup (R235Q and intronic induced neuron networks). Notably, this clustering paralleled patient clusters A and B observed at the proteomic level.

## Discussion

In this study, we demonstrated in patient-derived neurons that reduced MUNC18-1 protein levels were a shared cellular phenotype among all 6 patients with *STXBP1*-RD using 3 independent approaches (Western blotting, immunocytochemistry, and proteomics). This confirms earlier conclusions in mouse primary neurons and heterologous cells that missense as well as truncating variants in *STXBP1* result in lower cellular MUNC18-1 levels (13, 14, 15,43). Together with the notion that *STXBP1* variants range across the entire gene, without apparent hotspots and no robust correlations to clinical symptoms (4), we conclude that haploinsufficiency is the primary disease mechanism in *STXBP1*-RD. Furthermore, this study shows that patient neurons have substantially altered synaptic proteomes and that network properties are altered in patient neurons. Finally, this study provides the first indications for patient subgroup stratification, which interestingly does not align with patient mutation type (e.g., missense vs. loss-of-function) (Figure 4G–I) or specific functional domains in the sequence (Figure 1A).

In addition to the reduced MUNC18-1 levels, patient neurons present many more changes in protein levels. First, the upregulation of RNA processing proteins may relate to the degradation of incorrect *STXBP1* RNA, as proposed before (44). Second, the lower expression of the interaction partners of MUNC18-1 and other proteins involved in the synaptic vesicle cycle may be a direct consequence of the reduced MUNC18-1 levels. Finally, several other synaptic proteins were upregulated, most prominently RAPGEF4, a protein also upregulated during paradigms that increase synaptic strength (45,46); its effectors RAP1A and RAP1B; and other synaptic proteins associated with synaptic strength modulation, including ITGB1, ARL8, FLOT1, and MPP2 (47, 48, 49, 50). Hence, it seems plausible that compensatory mechanisms are activated to counteract the reduced MUNC18-1 levels and the functional consequences thereof.

Indeed, no synaptic deficits were observed in single patient neurons. This is in line with previous findings that single heterozygous mouse neurons (39) and heterozygous mouse neurons expressing *STXBP1* variants did not reveal deficits in basal synaptic transmission (13), although heterozygous mouse neurons showed small deficits during train stimulation (39). Thus, it is likely that homeostatic mechanisms succeed in compensating for the reduced MUNC18-1 levels in single neuron physiology. However, networks of patient neurons showed altered frequency, increased burst irregularity, and impaired synchronization. This is again in line with previous findings that networks of MUNC18-1 heterozygous neurons derived from embryonic stem cells have impaired neurotransmitter release (19), reduced spikes and bursts (21), and reduced network synchronicity and increased asynchronous firing of individual neurons (20). Hence, cellular phenotypes appear to be less penetrant in single neurons than in neuronal networks. This is consistent with previous findings where experimental genetic perturbations produced different synaptic phenotypes between single (autaptic) mouse neurons and neuron pairs or networks (32,51,52). Hence, homeostatic mechanisms may compensate more successfully for cellular deficits caused by the reduction in MUNC18-1 levels in a more reduced (single neuron) setting, but less so in a network setting, maybe also because in the current experiments, networks consisted of NGN2-induced excitatory neurons only, which is expected to trigger strong homeostatic responses due to the absence of inhibition. In patient-derived neurons, the observed network effects became even more pronounced on further increasing neuronal excitability, presumably exacerbating the extent to which MUNC18-1 haploinsufficiency is rate limiting for network function. Extrapolating this concept to even more complex settings, e.g., the intact patient brain, it is plausible that compensatory mechanisms fail to an increasing extent with increasing complexity, causing the clinical symptoms of *STXBP1*-RD.

This is the first study to our knowledge using a patient cohort of iPSC-derived neurons to investigate molecular/cellular disease mechanisms in SNAREopathies (3). For other SNAREopathy genes, previous studies have expressed disease variants in (isogenic) murine neurons. The majority of these variants in *SYT1*, *SNAP25*, *VAMP2*, *STX1*, and *UNC13A* do not affect cellular protein levels, but exhibit variant-specific deficits, which generally scale with clinical severity in patients (6,53, 54, 55, 56, 57). Moreover, for *SYT1*, *SNAP25*, and *VAMP2* variants, deficits are also observed when overexpressed on a wild-type background, indicating dominant negative activity and excluding a haploinsufficiency scenario as observed for *STXBP1* variants (6,53,54,57). These observations suggest that the primary disease mechanisms vary among SNAREopathies. Differences in experimental approaches and lack of models composed of patients’ own cells hinder direct comparison of primary and downstream functional phenotypes. Nevertheless, as all SNAREopathy variants are expected to affect the same core synaptic vesicle release machinery, it is conceivable that variants from different genes, with different primary effects on synaptic transmission, converge on common downstream mechanisms such as the incomplete homeostatic compensation at the network level that lead to impaired network synchronicity and regularity. More broadly, impairments in neuronal network synchronicity and regularity have been observed in in vitro models of other neurodevelopmental disorders (41,58, 59, 60, 61). Deficits in neuronal connectivity, excitability, regulation of specific synaptic proteins such as L-type voltage-gated channels, and alterations in energy metabolism all are associated with network asynchronicity and irregularity (58,60,62). Thus, although genes associated with neurodevelopmental disorders have diverse cellular functions, disease-associated variation in these genes appears to converge on deficits in neuronal network dynamics. Incomplete homeostatic compensation at the network level is a plausible final common pathway for all these cases.

### iPSC Disease Modeling to Identify Shared and Patient-Specific Pathobiology

Disease modeling using iPSCs allows studying both shared disease principles and patient-specific effects. Indeed, within the shared disease mechanism of *STXBP1*-RD proposed here, patient lines showed patient-specific effects in most experiments. Two patient clusters were distinguishable in proteomics (cluster A and cluster B) and in network activity (participation and frequency jump subgroups). Strikingly, these 2 subgroups largely overlapped. Although the current sample is too small to draw definitive conclusions, this overlap clearly suggests phenotypic subgroups. Clustering was not driven by variant type, as expected in haploinsufficiency. Most likely, the genetic background in each patient contributes to distinctive pathobiology, and additional genetic factors play a substantial role in disease etiology. Such modifier genes have been proposed for other Mendelian disorders, such as monogenic cases of autism spectrum disorder, *SCN1A*-related epilepsy, and cystic fibrosis (63, 64, 65). Induced neurons expressing a missense variant (D262V) showed by far the fewest significant proteome changes (Figure 4G) and clusters close to the control lines in PC analysis (Figure 4A, albeit the clinical phenotype is strong; Figure 1C). Cases of this variant type (missense mutations) may segregate from the heterogeneous group of loss-of-function mutations.

This discovery study identifies the cellular assays that are most likely to discern pathogenic hallmarks, thus providing a starting point for further mechanistic investigations into shared and distinct pathobiological mechanisms, cellular stratification of patient groups, and eventually cell-based diagnostics. Moreover, cellular models composed of patients’ own cells are invaluable to evaluate promising novel treatment strategies, such as the recently identified pharmacological chaperones that are proposed to stabilize MUNC18-1 (66). An in vitro model based on an individual patient’s own cells will allow selection of existing treatments tailored to the patient [e.g., as demonstrated previously for individuals harboring *SCN8A* variants (61)], greatly shortening the time to develop the optimal treatment regimen.

## Supplementary Material

Supplementary Material, incl Table S1-S3 and S5

Supplementary Table S4

Key Resources Table

## References

[1] Südhof T.C. (2014). The molecular machinery of neurotransmitter release (Nobel lecture). Angew Chem Int Ed Engl.

[2] Südhof T.C., Rothman J.E. (2009). Membrane fusion: Grappling with SNARE and SM proteins. Science.

[3] Verhage M., Sørensen J.B. (2020). SNAREopathies: Diversity in mechanisms and symptoms. Neuron.

[4] Stamberger H., Nikanorova M., Accorsi P., Angriman M., Benkel-Herrenbrueck I., Capovilla G. (2016). A neurodevelopmental disorder including epilepsy. Neurology.

[5] Schubert J., Siekierska A., Langlois M., May P., Huneau C., Becker F. (2014). Mutations in STX1B, encoding a presynaptic protein, cause fever-associated epilepsy syndromes. Nat Genet.

[6] Baker K., Gordon S.L., Melland H., Bumbak F., Scott D.J., Jiang T.J. (2018). SYT1-associated neurodevelopmental disorder: A case series. Brain.

[7] Deciphering Developmental Disorders Study (2017). Prevalence and architecture of de novo mutations in developmental disorders. Nature.

[8] López-Rivera J.A., Pérez-Palma E., Symonds J., Lindy A.S., McKnight D.A., Leu C. (2020). A catalogue of new incidence estimates of monogenic neurodevelopmental disorders caused by de novo variants. Brain.

[9] Verhage M., Maia A.S., Plomp J.J., Brussaard A.B., Heeroma J.H., Vermeer H. (2000). Synaptic assembly of the brain in the absence of neurotransmitter secretion. Science.

[10] Rizo J., Südhof T.C. (2012). The membrane fusion enigma: SNAREs, Sec1/Munc18 proteins, and their accomplices—guilty as charged?. Annu Rev Cell Dev Biol.

[11] Lammertse H.C.A., Van Berkel A.A., Iacomino M., Toonen R.F., Striano P., Gambardella A. (2020). Homozygous STXBP1 variant causes encephalopathy and gain-of-function in synaptic transmission. Brain.

[12] Saitsu H., Hoshino H., Kato M., Nishiyama K., Okada I., Yoneda Y. (2011). Paternal mosaicism of an STXBP1 mutation in OS. Clin Genet.

[13] Kovačević J., Maroteaux G., Schut D., Loos M., Dubey M., Pitsch J. (2018). Protein instability, haploinsufficiency, and cortical hyper-excitability underlie STXBP1-encephalopathy. Brain.

[14] Guiberson N.G.L., Pineda A., Abramov D., Kharel P., Carnazza K.E., Wragg R.T. (2018). Mechanism-based rescue of Munc18-1 dysfunction in varied encephalopathies by chemical chaperones. Nat Commun.

[15] Saitsu H., Kato M., Mizuguchi T., Hamada K., Osaka H., Tohyama J. (2008). De novo mutations in the gene encoding STXBP1 (MUNC18-1) cause early infantile epileptic encephalopathy. Nat Genet.

[16] Yamashita S., Chiyonobu T., Yoshida M., Maeda H., Zuiki M., Kidowaki S. (2016). Mislocalization of syntaxin-1 and impaired neurite growth observed in a human iPSC model for -related epileptic encephalopathy. Epilepsia.

[17] Miyamoto H., Tatsukawa T., Shimohata A., Yamagata T., Suzuki T., Amano K. (2019). Impaired cortico-striatal excitatory transmission triggers epilepsy. Nat Commun.

[18] Chen W., Cai Z.L., Chao E.S., Chen H., Longley C.M., Hao S. (2020). Stxbp1/Munc18-1 haploinsufficiency impairs inhibition and mediates key neurological features of STXBP1 encephalopathy. Elife.

[19] Patzke C., Han Y., Covy J., Yi F., Maxeiner S., Wernig M., Södhof T.C. (2015). Analysis of conditional heterozygous STXBP1 mutations in human neurons. J Clin Invest.

[20] Sun Z., Südhof T. (2021). A simple Ca^2+^-imaging approach to neural network analyses in cultured neurons. J Neurosci Methods.

[21] Ichise E., Chiyonobu T., Ishikawa M., Tanaka Y., Shibata M., Tozawa T. (2021). Impaired neuronal activity and differential gene expression in STXBP1 encephalopathy patient iPSC-derived GABAergic neurons. Hum Mol Genet.

[22] Kreitzer F.R., Salomonis N., Sheehan A., Huang M., Park J.S., Spindler M.J. (2013). A robust method to derive functional neural crest cells from human pluripotent stem cells. Am J Stem Cells.

[23] Brunner J.W., Lammertse H.C.A., van Berkel A.A., Koopmans F., Li K.W., Smit A.B. (2023). Power and optimal study design in iPSC-based brain disease modelling. Mol Psychiatry.

[24] Nadadhur A.G., Alsaqati M., Gasparotto L., Cornelissen-Steijger P., van Hugte E., Dooves S. (2019). Neuron-glia interactions increase neuronal phenotypes in tuberous sclerosis complex patient iPSC-derived models. Stem Cell Reports.

[25] Nadadhur A.G., Melero J.E., Meijer M., Schut D., Jacobs G., Wan Li K. (2017). Multi-level characterization of balanced inhibitory-excitatory cortical neuron network derived from human pluripotent stem cells. PLoS One.

[26] Zhang Y., Pak C., Han Y., Ahlenius H., Zhang Z., Chanda S. (2013). Rapid single-step induction of functional neurons from human pluripotent stem cells. Neuron.

[27] Nehme R., Zuccaro E., Ghosh S.D., Li C., Sherwood J.L., Pietilainen O. (2018). Combining NGN2 programming with developmental patterning generates human excitatory neurons with NMDAR-mediated synaptic transmission. Cell Rep.

[28] Meijer M., Rehbach K., Brunner J.W., Classen J.A., Lammertse H.C.A., van Linge L.A. (2019). A single-cell model for synaptic transmission and plasticity in human iPSC-derived neurons. Cell Rep.

[29] Gonzalez-Lozano M.A., Koopmans F. (2019). Data-independent acquisition (SWATH) mass spectrometry analysis of protein content in primary neuronal cultures. Neuromethods.

[30] Hjorth J.J., Dawitz J., Kroon T., Pires J., Dassen V.J., Berkhout J.A. (2016). Detection of silent cells, synchronization and modulatory activity in developing cellular networks. Dev Neurobiol.

[31] Wang R., Wang Z., Wang J., Li S. (2019). SpliceFinder: Ab initio prediction of splice sites using convolutional neural network. BMC Bioinformatics.

[32] Van Berkel A.A., Koopmans F., Gonzalez-Lozano M.A., Lammertse H.C.A., Feringa F., Bryois J. (2022). Dysregulation of synaptic and developmental transcriptomic/proteomic profiles upon depletion of MUNC18-1. eNeuro.

[33] Koopmans F., van Nierop P., Andres-Alonso M., Byrnes A., Cijsouw T., Coba M.P. (2019). SynGO: An evidence-based, expert-curated knowledge base for the synapse. Neuron.

[34] Wierda K.D., Toonen R.F., de Wit H., Brussaard A.B., Verhage M. (2007). Interdependence of PKC-dependent and PKC-independent pathways for presynaptic plasticity. Neuron.

[35] Meijer M., Cijsouw T., Toonen R.F., Verhage M. (2015). Synaptic effects of Munc18-1 alternative splicing in excitatory hippocampal neurons. PLoS One.

[36] Meijer M., Dörr B., Lammertse H.C., Blithikioti C., van Weering J.R., Toonen R.F. (2017). Tyrosine phosphorylation of Munc18-1 inhibits synaptic transmission by preventing SNARE assembly. EMBO J.

[37] Classen J., Saarloos I., Meijer M., Sullivan P.F., Verhage M. (2020). A Munc18-1 mutant mimicking phosphorylation by Down syndrome-related kinase Dyrk1a supports normal synaptic transmission and promotes recovery after intense activity. Sci Rep.

[38] Schmitz S.K., King C., Kortleven C., Huson V., Kroon T., Kevenaar J.T. (2016). Presynaptic inhibition upon CB 1 or mG lu2/3 receptor activation requires ERK/MAPK phosphorylation of Munc18-1. EMBO J.

[39] Toonen R., Wierda K., Sons M.S., de Wit H., Cornelisse L.N., Brussaard A. (2006). Munc18-1 expression levels control synapse recovery by regulating readily releasable pool size. Proc Natl Acad Sci U S A.

[40] Combrisson E., Jerbi K. (2015). Exceeding chance level by chance: The caveat of theoretical chance levels in brain signal classification and statistical assessment of decoding accuracy. J Neurosci Methods.

[41] Frega M., Linda K., Keller J.M., Gümüş-Akay G., Mossink B., van Rhijn J.R. (2019). Neuronal network dysfunction in a model for Kleefstra syndrome mediated by enhanced NMDAR signaling. Nat Commun.

[42] Frega M., van Gestel S.H., Linda K., van der Raadt J., Keller J., Van Rhijn J.R. (2017). Rapid neuronal differentiation of induced pluripotent stem cells for measuring network activity on micro-electrode arrays. J Vis Exp.

[43] Martin S., Papadopulos A., Collins B.M., Meunier Correspondence F.A. (2014). Increased polyubiquitination and proteasomal degradation of a Munc18-1 disease-linked mutant causes temperature-sensitive defect in exocytosis. Cell Rep.

[44] Saitsu H., Kato M., Matsumoto N. (2012). Haploinsufficiency of STXBP1 and Ohtahara syndrome. Epilepsia.

[45] Gloerich M., Bos J.L. (2010). Epac: Defining a new mechanism for cAMP action. Annu Rev Pharmacol Toxicol.

[46] Fernandes H., Riordan S., Nomura T., Remmers C.L., Kraniotis S., Marshall J.J. (2015). Epac2 mediates cAMP-dependent potentiation of neurotransmission in the hippocampus. J Neurosci.

[47] Vukoja A., Rey U., Petzoldt A.G., Lipowsky R., Sigrist S.J., Haucke V. (2018). Presynaptic biogenesis requires axonal transport of lysosome-related vesicles. Neuron.

[48] Chan C.S., Weeber E.J., Zong L., Fuchs E., Sweatt J.D., Davis R.L. (2006). β1-Integrins are required for hippocampal AMPA receptor-dependent synaptic transmission, synaptic plasticity, and working memory. J Neurosci.

[49] Kim G., Luján R., Schwenk J., Kelley M.H., Aguado C., Watanabe M. (2016). Membrane palmitoylated protein 2 is a synaptic scaffold protein required for synaptic SK2-containing channel function. Elife.

[50] Swanwick C.C., Shapiro M.E., Vicini S., Wenthold R.J. (2010). Flotillin-1 promotes formation of glutamatergic synapses in hippocampal neurons. Dev Neurobiol.

[51] Liu H., Dean C., Arthur C.P., Dong M., Chapman E.R. (2009). Autapses and networks of hippocampal neurons exhibit distinct synaptic transmission phenotypes in the absence of synaptotagmin I. J Neurosci.

[52] Wierda K.D., Sørensen J.B. (2014). Innervation by a GABAergic neuron depresses spontaneous release in glutamatergic neurons and unveils the clamping phenotype of synaptotagmin-1. J Neurosci.

[53] Alten B., Zhou Q., Shin O.H., Esquivies L., Lin P.Y., White K.I. (2021). Role of aberrant spontaneous neurotransmission in SNAP25-associated encephalopathies. Neuron.

[54] Bradberry M.M., Courtney N.A., Dominguez M.J., Lofquist S.M., Knox A.T., Sutton R.B., Chapman E.R. (2020). Molecular basis for synaptotagmin-1-associated neurodevelopmental disorder. Neuron.

[55] Lipstein N., Verhoeven-Duif N.M., Michelassi F.E., Calloway N., van Hasselt P.M., Pienkowska K. (2017). Synaptic UNC13A protein variant causes increased neurotransmission and dyskinetic movement disorder. J Clin Invest.

[56] Vardar G., Gerth F., Schmitt X.J., Rautenstrauch P., Trimbuch T., Schubert J. (2020). Epilepsy-causing STX1B mutations translate altered protein functions into distinct phenotypes in mouse neurons. Brain.

[57] Simmons R.L., Li H., Alten B., Santos M.S., Jiang R., Paul B. (2020). Overcoming presynaptic effects of VAMP2 mutations with 4-aminopyridine treatment. Hum Mutat.

[58] Alsaqati M., Heine V.M., Harwood A.J. (2020). Pharmacological intervention to restore connectivity deficits of neuronal networks derived from ASD patient iPSC with a TSC2 mutation. Mol Autism.

[59] Gutierrez R.C., Hung J., Zhang Y., Kertesz A.C., Espina F.J., Colicos M.A. (2009). Altered synchrony and connectivity in neuronal networks expressing an autism-related mutation of neuroligin 3. Neuroscience.

[60] Klein Gunnewiek T.M., Van Hugte E.J.H., Frega M., Guardia G.S., Foreman K., Panneman D. (2020). m.3243A > G-induced mitochondrial dysfunction impairs human neuronal development and reduces neuronal network activity and synchronicity. Cell Rep.

[61] Tidball A.M., Lopez-Santiago L.F., Yuan Y., Glenn T.W., Margolis J.L., Clayton Walker J. (2020). Variant-specific changes in persistent or resurgent sodium current in SCN8A-related epilepsy patient-derived neurons. Brain.

[62] Plumbly W., Brandon N., Deeb T.Z., Hall J., Harwood A.J. (2019). L-type voltage-gated calcium channel regulation of in vitro human cortical neuronal networks. Sci Reports.

[63] Gallati S. (2014). Disease-modifying genes and monogenic disorders: Experience in cystic fibrosis. Appl Clin Genet.

[64] Niemi M.E.K., Martin H.C., Rice D.L., Gallone G., Gordon S., Kelemen M. (2018). Common genetic variants contribute to risk of rare severe neurodevelopmental disorders. Nature.

[65] de Lange I.M., Mulder F., van’t Slot R., Sonsma A.C.M., van Kempen M.J.A., Nijman I.J. (2020). Modifier genes in SCN1A-related epilepsy syndromes. Mol Genet Genomic Med.

[66] Abramov D., Guiberson N.G.L., Daab A., Na Y., Petsko G.A., Sharma M., Burré J. (2021). Targeted stabilization of Munc18-1 function via pharmacological chaperones. EMBO Mol Med.

