# Erratum

**DOI:** 10.1016/j.bpsgos.2023.11.003

**Published:** 2023-11-17

Erratum to: “Reduced MUNC18-1 Levels, Synaptic Proteome Changes, and Altered Network Activity in *STXBP1*-Related Disorder Patient Neurons,” by van Berkel *et al.* (*Biol Psychiatry Glob Open Sci*; https://doi.org/10.1016/j.bpsgos.2023.05.004).

A correction for this article has become necessary, as the mutation originally reported as K308X for patient 5 should instead be R235Q. Specifically, authors of a previous study, on which this study was partially built, recently found that the *STXBP1* mutation pathogenic for this patient is c.704G>A, p.Arg235Gln, and not c.[922A>T], p.[Lys308(∗)]. According to the authors, the reporting mistake in the previous article was caused by a mix-up between the mutations of two different patients affected by the same condition.

All references to this mutation have now been corrected to R235Q.

