# Supplementary material for: Reduced MUNC18-1 Levels, Synaptic Proteome Changes, and Altered Network Activity in STXBP1-Related Disorder Patient Neurons
Source: Biol Psychiatry Glob Open Sci. 2023 May 30;4(1):284–98. doi: 10.1016/j.bpsgos.2023.05.004 (PMC10829628; doi:10.1016/j.bpsgos.2023.05.004)
Supplement: Supplementary Material, incl Table S1-S3 and S5 [file mmc1.pdf]

SUPPLEMENTARY INFORMATION

Reduced MUNC18-1 Levels, Synaptic Proteome Changes, and Altered Network Activity in STXBP1-Related Disorder Patient Neurons

Van Berkel *et al.*

Supplemental Materials & Methods

Compliance with ethical guidelines and legislation

Sampling of skin biopsies and collection of clinical information was approved by the Medical Ethics Review Committee of the Amsterdam University Medical Centre, in compliance with all relevant legislation. Informed consent was obtained from the parents/legal guardians prior to inclusion in the present study. For one patient (carrying a R235Q variant), a case report had already been published [1] from which the clinical information was obtained. The skin biopsy from this patient was obtained prior to the present study for clinical/diagnostic purposes and shared by the principal physician.

Glia feeder layers were generated from new born Wistar rat pups (Crl:WI, strain code 003). Animals were housed and handled according to the Institutional and Dutch governmental guidelines.

Patient phenotyping

For all STXBP1-related disorder patients with exception of the R235Q case (see above), clinical phenotyping was performed using a questionnaire to be filled out by the parents/caregivers. In addition, most of the patients were also assessed by a clinical geneticist and a neurologist for additional clinical assessments.

Skin biopsy and fibroblast culture

Skin biopsies were performed under sterile conditions after local anaesthesia with 2% lidocaine with adrenaline, using a 3 mm skin punch. The biopsy was cut in smaller pieces and transferred to culture flasks containing fibroblast medium (Ham's F10 medium (Sigma-Aldrich), 5% FBS, 2% Ultrocer G, 1% P/S) to grow a monolayer fibroblast culture. Medium was refreshed after one week, and subsequently refreshed twice a week. Confluent cultures were passaged using Trypsin-EDTA (Sigma Aldrich). Genomic integrity of fibroblast cultures was assessed based on SNP analysis (see below), and fibroblast medium was tested against mycoplasma contamination.

We have used three healthy control iPSC lines in this study. Details can be found in the Table S5.

iPSC generation and culture

Fibroblast cultures were transduced using non-integrating Sendai virus containing Yamanaka factors Oct3/4, Sox2, Klf4 and c-Myc (CytoTune - iPSC 2.0 Sendai Reprogramming Kit (Invitrogen, #A16517)). After seven days, transduced cells were dissociated with 0.25% Trypsin-EDTA and replated on vitronectin-coated culture dishes in fibroblast medium. Medium was changed to Essential 8 Medium (GIBCO, Thermo Fisher) after 24 hours, and replaced every day until colonies of appropriate size emerged (three to four weeks). Single iPSC colonies were picked, transferred to 96-well vitronectin-coated culture plates and expanded to multiple 6-wells. Genomic integrity of clones was determined based on SNP analysis. Clones that passed quality control were passaged for 15 times to obtain vector-free iPSCs, after which genomic integrity was again assessed.

iPSC cultures were maintained in Essential E8 medium + 0.1% Pen/Strep (Invitrogen), on Matrigel-coated plates. When confluent, cells were dissociated using Gentle Cell Dissociation Reagent (GIBCO, Thermo Fisher) and replated in Essential E8 medium supplemented with 5µM ROCK Inhibitor (RI Y-27632; Tebu Bio). Genomic integrity of iPSC lines was periodically tested based on SNP arrays. In addition, cell cultures were regularly tested for mycoplasma contamination.

SNP analysis and CNV calling

DNA from cell cultures was isolated using ReliaPrep gDNA Tissue Miniprep System (Promega). Samples were processed by the Global Screening Array (GSA) Consortium Project at Erasmus MC Rotterdam, The Netherlands on the Illumina GSA beadchip GSA MD v1. SNP data was processed and annotated with Illumina Genome Studio software. *iPsychCNV* package was used for copy number variant (CNV) calling, which integrates B allele frequency distribution and Log R ratio to reduce false positive detection (Bertalan, 2017). CNVs larger than 500kB and containing more than 100 SNPs were flagged, and compared against gene lists associated with brain development and synapse GO terms. Only clones with no CNVs called were included in this study. Genomic resemblance of all processed samples was performed for every quality control round to confirm genomic identity using plink 2.0 [2]. Samples with an overlap of >90% SNPs were assumed to have the same genomic identity.

Neuronal induction

iPSCs were infected in suspension (in E8 + RI) with high-titer lentiviral particles encoding pTet-O-Ngn2-puro (Addgene #52047) and FUΔGW-rtTa (Addgene #19780). Infected iPSCs were expanded for a maximum of five passages. To start neuronal induction, E8 medium was replaced by N2-supplemented medium (DMEM/F12 medium, 200mM Glutamax, 20% Dextrose, 1% N2 supplement B and 0.1% Pen/Strep (Invitrogen) supplemented with doxycycline hyclate (2µg/ml, Sigma Aldrich) and dual SMAD inhibitors (100nM LDN193189, 10µM SB431542, 2µM XAV939, from Stemgent, Tebu-Bio and Sigma-Aldrich, respectively). On day 2, medium was refreshed, and puromycin (concentration determined separately for each iPSC line, 1-3 µg/ml, Merck-Millipore) was added. This step was repeated for another 24 hours. On day 4, cultures were refreshed with N2-supplemented medium with 10µM FUDR (Sigma-Aldrich). The next day, iNeurons were replated onto 18mm glial feeder layer coverslips (2k on microdot array or 50k on mass culture) or 300k on poly-L-ornithine/laminin-coated (POL; Sigma-Aldrich) 6 well-plates. The glial feeder layer was created by plating rat glia grown on either etched glass coverslips applied with 0.1mg/ml poly-D-lysine and 0.2 mg/ml rat tail collagen (BD Biosciences) for mass cultures, or on coverslips stamped with microdots of 0.1mg/ml poly-D-lysine (Sigma), 0.7mg/ml rat tail collagen and 10mM acetic acid for microdot cultures, as described previously [3]. Four days after replating, mass cultures were treated once with 2µM AraC (Sigma).

iNeurons were maintained in Neurobasal medium, supplemented with 200mM Glutamax, 20% Dextrose, NEAA, B27, 0.1% P/S, 0.5% Fetal bovine serum, 10ng/ml BDNF, 10ng/ml CNTF, 10ng/ml GDNF, 2µg/ml Doxycycline hyclate, at 37 degrees Celsius and 5% CO_2. Medium was replaced with 50% fresh medium once (glial microdot plates) or twice (mass cultures and POL plates) a week.

Immunocytochemistry

Neuronal cultures were fixed at DIV39-42 after doxycycline induction with 3.7% paraformaldehyde (PFA; Electron Microscopy Sciences) for 20min at room temperature (RT) for presynaptic and MUNC18-1 stainings, or with ice-cold Methanol for 10min at -20 Celsius for postsynaptic stainings. Next, neurons were washed three times with Phosphate Buffered Saline pH = 7.4 (PBS, Sigma-Aldrich). Cultures were permeabilized with 0.5% Triton X-100, followed by 30 minutes incubation in PBS + 0.1% Triton X-100 + 2% Normal Goat Serum (NGS) (Blocking solution) to block a-specific binding. Cultures were stained with primary antibodies for 2 hours at RT, diluted in blocking solution. The following antibodies were used: chicken anti-MAP2 (1:500, Abcam), guinea pig anti-Synaptophysin-1 (1:1000, Synaptic Systems #101004), mouse-PSD-95 (1:500, Abcam), rabbit-MUNC18-1 (1:200, Sigma HPA023483). This antibody detects both splice variants of MUNC18-1, epitope: NGITEENLNKLIQHAQIPPEDSEIITNMAHLGVPIVTDSTLRRRSKPERKERISEQTYQLSRWTPI IKDIMEDTIEDKLDTKHYPYISTRSSASFSTTAVSARYGHWHKNKAPGEYRSGP. Following three washes with PBS, cultures were incubated with secondary antibodies Alexa Fluor (1:1000, Invitrogen) for 1 hour at RT. After three additional washes, coverslips were mounted on microscopic slides with Mowiol-DABCO.

Images were acquired on a NIKON Ti-Eclipse microscope, equipped with confocal scanner model A1R+, using a 40X oil immersion objective (NA=1.3, Carl Zeiss). Confocal settings were optimized for every culture batch, and Z stacks were acquired with 0.5 um intervals. For analysis, Z stacks were collapsed to maximal projections. Neuronal and synapse morphology (on microdot arrays) were analysed in MATLAB with the build-in software SynD [4]. Synapse detection settings were kept the same between cultures. For MUNC18-1 stainings (mass cultures), image analysis was performed in ImageJ software. MUNC18-1 fluorescence intensity was measured in MAP2-positive areas (Dendrites), MUNC18-1-positive areas (Total neuron) and synaptophysin-1-positive areas (Synapses). Intensity values were normalized to the culture batch-corresponding geomean of the control lines.

Western blot

DIV39-42 neuronal cultures on POL-coated plates were washed once with ice-cold PBS and collected in ice-cold PBS supplemented with EDTA-free PI (Roche). Cells were pelleted by 5 min centrifuge at 3000rcf at 1 degree Celsius, and diluted in SDS sample buffer. Samples were heated at 90 Celsius for 5 min and further homogenized using an insulin syringe (BD MicroFine). Proteins were separated on 10% SDS-PAGE and transferred to nitrocellulose membranes. Next, membranes were incubated with PBS + 2% BSA (Thermo Fisher Scientific) + 0.05% Tween-20 (Sigma) on an orbital shaker for 1 hour at RT, followed by an overnight incubation with primary antibodies, diluted in blocking solution. The following antibodies were used: mouse anti-MUNC18-1 (1:1000, Transduction Labs), rabbit anti-MUNC18-1 (1:1000, 2701 [5]), mouse anti-Syntaxin-1 (1:5000, Sigma) and mouse anti-gamma-tubulin (1:1000, Sigma). Next day, membranes were washed in PBS for three times and stained with secondary antibodies IRDye 680LT anti mouse and IRDye 800CW anti rabbit (1:5000, LI-COR), diluted in PBS+0.05% Tween for 30 min at RT. Following three additional washes, membranes were scanned by an Odyssey CLx Imaging System (LI-COR) and analysed with Image Studio Lite software (LI-COR).

qPCR

DIV39-42 neuronal cultures on POL-coated plates were washed once in ice-cold PBS, collected in TRIzol reagent (Life Technologies) and stored at -80 Celsius for further use. To isolate total RNA, samples were incubated in TRIzol in phase-lock gel tubes (QuantaBio) for 5 min at RT. Next, chloroform was added to a final concentration of 20%, and tubes were shaken vigorously for 15 sec. Samples were centrifuged at 11500 rpm for 15 min at 4 degrees Celsius, and the aqueous phase was transferred to new tubes. Equal amount of isopropanol + 0.4% Glycoblue Dye (Life Technologies) was added to precipitate the sample for 10 min at RT. After the samples were centrifuged for 10 min at 1.000rpm at 4 degrees Celsius, 75% Ethanol was added to the pellet. Next, the samples were centrifuged for 5 min at 750rpm at 4 degrees Celsius. All Ethanol was removed and the pellet was diluted in RNAse-free water.

RNA purity and quantity were assessed on a NanoDrop spectrophotometer. 500ng RNA was reversed transcribed into cDNA with sensiFAST cDNA Synthesis kit (Bioline) according to manufacturer's instructions. Next, cDNA was quantified using SensiFAST SYBR No-ROX (Bioline) in a LightCycler 480 (Roche Life Sciences), using 10μM primers that detect both known splice variants of *stxbp1*, as shown in Table S3. The following program was used: 5 min incubation at 95 degrees Celsius, 4.8C/s ramp rate, followed by 50 cycles of 10 sec 95 degrees Celsius (4.8 C/s), 20 sec 60 degrees Celsius (2.4 C/s), 1 sec 72 degrees Celsius (4.8 C/s). Primers showed efficiencies between 90-110% and clean melting curves. Cp values were determined using the second derivative maximum method. Samples were quantified in triplicates and average values were used. RNA levels were normalized to MYC and GAPDH. D262V did not reach the required number of independent observations and was therefore excluded from this analysis.

Mass spectroscopy

Cells were washed 2 times with ice cold PBS. 500 μL protease inhibitor (PI) solution in PBS (complete EDTA-free protease inhibitor tablets, Roche 05056489001) was added to each well. Cells were collected by gentle scraping and spinned down for 5 minutes at 3000 rcf at 4C. Supernatant was removed and the pellet was resuspended in 20μL loading buffer (4% SDS, 100mM Tris pH 6.8, 0.04% bromophenol blue, 200mM DTT, 20% glycerol, and PI in PBS). Samples were snap frozen and stored at -80C until further processed. An SDS–PAGE LC-MS/MS approach was used for protein identification as described previously [6]. The Uniprot human reference proteome database (SwissProt+ TrEMBL, version 2019-11) was used to annotate spectra. SWATH data were searched against a spectral library (peptides and proteins identified from DDA data by MaxQuant) of DIV15 and DIV42 NGN2-neurons and rat glia, using Spectronaut 13.7 [7] with default settings. The resulting abundance values and qualitative scores for each peptide in the spectral library were exported for further analysis.

MS proteomics data analyses were performed using R language for statistical computing. MS-DAP 0.2.6.4 (https://github.com/ftwkoopmans/msdap) was used for the interpretation of data quality and differential expression analysis (DEA). While importing the Spectronaut data report, fragment group MS2 total peak areas without Spectronaut normalization were selected to represent peptide intensity values and both proteins from the MaxQuant contaminant database and iRT peptides were removed from the dataset.

In each statistical contrast, peptides observed in both sample groups with Spectronaut confidence score <= 0.01 in at least 2 samples (biological replicates) were selected. MS-DAP's mode-between normalization was then applied to this data subset and finally the MSqRob statistical model was used for differential testing with "culture batch" as random variable [8]. The significance threshold was set at 1% FDR. All data visualizations and MS-DAP parameters are included in the MS-DAP report.

Functional annotation and enrichment of significant regulated proteins was done in Cytoscape plug-in ClueGO [9], using the Biological Process Gene Ontology (GO) database updated on 10-02-2021. ClueGO analyses were performed including the following settings: Biological Process, GO term grouping, GO tree interval was set 0-20 (all levels), GO terms consisting of min. five genes and min. 5% of the term. For Fig. 4A and D, patient-specific contrasts (regulated proteins at 1% FDR between controls and patient X) were loaded as independent lists and analyzed together. To prioritize GO terms for this analysis, all detected proteins were used as background and only GO terms enriched at $p<0.1$ were included. Functional annotation of synaptic proteins was done in the SynGO portal (https://syngoportal.org, and [9]). Sunburst plot was made visualizing all significantly regulated proteins colour-coded by gene count of each specific term including child terms. Heatmap visualizations were done in RStudio using the *gplots* package. Hierarchical clustering was performed using the ward.D method and euclidean distance. Similarity matrix was made in RStudio. For every STXBP1-related disorder pair, proteins were included that were significantly (qvalue set at 0.001) regulated in either iNeuron line. Proteins with fold changes of zero in either iNeuron line were excluded, as MSqRob model uses shrinkage by ridge regression to assign zero-fold change to proteins for which the model is highly confident the null hypothesis cannot be rejected [8]. Below the diagonal in the similarity matrix (Fig. 5B), fold changes of proteins in each contrast were visualized, and a robust regression fit using the *MASS* package (fit method = MM). D207G did not reach the required number of independent observations and was therefore excluded from this analysis.

Electrophysiology

Synapse physiology at single-neuron resolution was recorded by whole-cell patch-clamp electrophysiology of autaptic iNeurons recorded at DIV39-42. A Multiclamp 700B amplifier (Molecular Devices) with Digidata 1440A/1550B and Clampex 10 software (Molecular Devices) was used. Recordings were performed at room temperature, using external solution containing 140mM NaCl, 2.4mM KCl, 2mM $CaCl_2$, 4mM $MgCl_2$, 10mM HEPES, 10mM glucose (pH adjusted to 7.3 with NaOH; 300mOsm). Borosilicate glass pipettes (2.5-4.5MOhm) were used with internal solution (136mM KCl, 17.8mM HEPES, 1mM EGTA, 0.6mM $MgCl_2.6H_2O$, 4mM ATP-Mg, 0.3mM GTP-Na, 12mM phosphocreatine dipotassium salt, 50 units/mL phosphocreatine kinase, pH 7.3, osmolarity ~300mOsm). Synaptic currents were measured in whole-cell voltage-clamp configuration at a holding potential of -70mV. First, spontaneous activity was recorded with a sampling frequency of 20kHz (Bessel filter 5-6kHz), followed by a series of stimulations recorded with sampling frequency of 10kHz (Bessel filter 2kHz). Action potentials were elicited by a 1ms step to +30mV from the holding potential. First, the first evoked EPSC was recorded, followed by a paired-pulse stimulation (50ms inter-pulse interval) and a series of train stimulations of 5 pulses at 5Hz, 10Hz and 20Hz, with 90 seconds rest in between each protocol, to induce synaptic depression. Each train was followed after 2 seconds with a single stimulation pulse to assess the recovery after synaptic depression. Lastly, 80 action potentials at 40Hz frequency were given to fully deplete the readily releasable pool. Recovery of the pool was assessed by two single pulses, at 2 seconds and 60 seconds after the train.

Recordings were accepted for analysis if the leak current did not exceed -400pA and series resistance remained below 15 MΩ. Furthermore, traces were analyzed for 'atypical' (multi-peaked) synaptic responses. For each neuron, the first evoked EPSC was used to quantify the relative contribution of additional peaks (total deviation ratio or TDR; see Brunner, Lammertse, van Berkel et al. [11]. Concurrently, an experienced user visually inspected each recording to reach an include/exclude decision. Plotting a histogram of TDR values for the entire dataset split by user decision (Fig. S3) showed TDR 0.15 as cut-off criterion for which relatively more 'include-recordings' than 'exclude-recordings' would be taken along. All neurons with a TDR below 0.15 were therefore

excluded from further analysis, whereas if the first EPSC met the criteria, the neuron would be taken along for all downstream analyses (i.e., no separate selection was performed for each synaptic parameter).

All offline analysis was performed using in-house developed Matlab scripts (available via https://github.com/vhuson/viewEPSC) and Clampfit (Molecular Devices, v10.7). Size of the RRP was estimated by back-extrapolation (Neher, 2015) fitted to the last 20 pulses of the train based on the total charge.

Calcium imaging

DIV39-42 mass cultures were incubated for 8 min at 37 degrees Celsius, 5% CO_2 in 2µM Fluo-4, AM (Thermo Fisher, #F14201) in Neurobasal medium. Next, medium was replaced with Tyrode's solution (119mM NaCl, 2.5mM KCl_2, 2mM $CalCl_2$, 2mM $MgCl_2$, 25mM HEPES, 20mM Glucose, pH 7.4, mOsmol 280), and coverslips were transferred to a NIKON Ti-Eclipse microscope equipped with a Confocal A1R (LU4A Laser) unit, an EMCCD camera (Andor DU-897), appropriate filter sets, x10 air (NA = 0.45) objective, and controlled by NisElements 4.30 software. Coverslips were perfused with Tyrode's solution. DIC images were taken for ROI detection. Baseline time-lapse recordings (8Hz; exposure time 124ms) were acquired for 5 min at RT. Subsequently, perfusion was switched to Tyrode's solution supplemented with 100µM potassium agonist 4-Aminopyridine (Sigma). After an incubation of 2 min, networks were recorded for another 5 min at 8Hz.

Network activity was analyzed using 'Event Detection Analysis' (EvA) with in-house Matlab software scripts [10]. In short, ROIs of neuronal somas were manually created based on the DIC image. Calcium events were detected using an iterative method to set the detection threshold. To detect synchronous network events, binarized traces of individual neurons were aligned and all peaks of summed events that passed an iteratively-defined threshold value (averaged 5 standard deviations above baseline from 500 scrambled traces) were marked as network events. The participation index of individual neurons is defined as the proportion of network events a particular neuron joins. The fraction of full participation is defined as the fraction of neurons within one network that participated in all network events (i.e. fraction of neurons with participation index of 1). CoV measurements were calculated as the standard deviation divided by the average within one network. Characteristics of events were defined as followed: Mean event amplitude = averaged peak amplitude of Fluo4-AM intensity of all neurons in the network; Mean event area = averaged area under the curve of Fluo4-AM intensity from start to end of event; Mean event rise time = averaged time from start event to peak amplitude; Mean event decay time = averaged time from peak amplitude to end event, Mean event duration = time from start to end of event. For more detailed information on network detection and parameter definitions, we refer to [10]. D262V did not reach the required number of independent observations and was therefore excluded from this analysis.

Linear Discriminant Analysis

LDA was performed in Rstudio (v.1.2.5042) using the *caret* and *MASS* packages. The datasets were split in 80% training and 20% test sets. LDA was performed twice; first to discriminate between control and STXBP1-related disorder observations (LDA - Condition), and second to discriminate between all iNeuron lines (LDA - Line). Accuracy was determined based on performance to discriminate test dataset. Variables were standardized before LDA was computed to use the discriminator weights as measures of variable importance. Linear Discriminants were plotted using the *ggplot2* package.

Statistical analyses

Outliers were defined as 3 standard deviations from the group mean and removed from datasets before statistical analyses were executed. Statistical comparisons were performed using linear mixed-effects models to account for the nested structure of the data, including information on which culture batch each observation belonged to was included as a covariate in the analysis. For each parameter, group-level differences were assessed by first fitting a model including information on line and batch, and subsequently a model including which group each line belongs to (i.e., 'Controls' or 'Patients'). Comparing the fit of these two models using a chi-square test allows to identify whether control and STXBP1-related disorder lines overarchingly differ from each other (hereafter referred to as 'group-level' comparisons). Subsequently, a similar procedure was carried out but comparing each STXBP1-related disorder line separately to the three control lines, to identify any line-specific differences that are not general to all six STXBP1-related disorder lines (hereafter referred to as 'patient-level' comparisons). Because of the explorative nature of the study, p-values between 0.1 and 0.05 were reported in the figures. Note that the p-values reported in the figures are not corrected for multiple testing. For each parameter, outcome after correction for multiple testing is reported in the Statistics Table S1. Statistical analysis was performed using R/RStudio, linear mixed-effects models were fitted using the *lme4* package. Graphs were made using GraphPad Prism 9 (GraphPad Software) or R/Rstudio (v. 1.2.5042). Statistical details are listed in Table S1. Sample size numbers are indicated in Figure legends; n represent number of observations, N represent number of independent culture batches (i.e. biological replicates). Data were plotted as boxplots with Tukey whiskers that extend to 1.5 times the inter-quartile range. In plots comparing controls and patients overarchingly, data points outside of this range are plotted as individual dots. In plots comparing groups separately, dots represent individual data points.

Supplemental References

1. Dilena R, Striano P, Traverso M, Viri M, Cristofori G, Tadini L, et al. Dramatic effect of levetiracetam in early-onset epileptic encephalopathy due to STXBP1 mutation. Brain Dev. 2016;38:128–131.
2. Chang CC, Chow CC, Tellier LC, Vattikuti S, Purcell SM, Lee JJ. Second-generation PLINK: rising to the challenge of larger and richer datasets. Gigascience. 2015;4:7.
3. Meijer M, Rehbach K, Brunner JW, Classen JA, Lammertse HCA, van Linge LA, et al. A Single-Cell Model for Synaptic Transmission and Plasticity in Human iPSC-Derived Neurons. Cell Rep. 2019;27:2199-2211.e6.
4. Schmitz SK, Hjorth JJJ, Joemai RMS, Wijntjes R, Eijgenraam S, de Bruijn P, et al. Automated analysis of neuronal morphology, synapse number and synaptic recruitment. J Neurosci Methods. 2011;195:185–193.
5. Cijsouw T, Weber JP, Broeke JH, Broek JAC, Schut D, Kroon T, et al. Munc18-1 redistributes in nerve terminals in an activity- and PKC-dependent manner. J Cell Biol. 2014;204:759–775.
6. Gonzalez-Lozano MA, Koopmans F. Data-Independent Acquisition (SWATH) Mass Spectrometry Analysis of Protein Content in Primary Neuronal Cultures. Neuromethods. 2019;146:119–127.
7. Bruderer R, Bernhardt OM, Gandhi T, Miladinović SM, Cheng LY, Messner S, et al. Extending the limits of quantitative proteome profiling with data-independent acquisition and application to acetaminophen-treated three-dimensional liver microtissues. Mol Cell Proteomics. 2015;14:1400–1410.
8. Goeminne LJE, Gevaert K, Clement L. Peptide-level robust ridge regression improves estimation, sensitivity, and specificity in data-dependent quantitative label-free shotgun proteomics. Mol Cell Proteomics. 2016;15:657–668.
9. Koopmans F, van Nierop P, Andres-Alonso M, Byrnes A, Cijsouw T, Coba MP, et al. SynGO: An Evidence-Based, Expert-Curated Knowledge Base for the Synapse. Neuron. 2019;103:217-234.e4.
10. Hjorth JJJ, Dawitz J, Kroon T, Pires J, Dassen VJ, Berkhout JA, et al. Detection of silent cells, synchronization and modulatory activity in developing cellular networks. Dev Neurobiol. 2016;76:357–374.
11. Brunner JW, Lammertse HCA, van Berkel AA, Koopmans F, Li KW, Smit AB, Toonen RF, Verhage M, van der Sluis S. Power and optimal study design in iPSC-based brain disease modelling. Mol Psychiatry. 2022; Epub ahead of print.

Supplemental table and figure legends

Suppl. Table. 1: Table with clinical information

Table summarizing the clinical phenotype of each STXBP1-RD patient included in this study. For patients 1-4 and P6, the primary caregivers filled in a questionnaire, patients 2, 3,4 and 6 underwent specific clinical assessment as part of this study by a paediatric neurologist and clinical geneticist. For patient 5 (R235Q), a case report was published (61) from which clinical information was obtained.

Suppl. Table 2: Table with statistical parameters

Table containing all statistical data for all parameters tested in this study. For each parameter, the sample size per iNeuron line is shown. The first row for each parameter shows the outcome of the χ^2 test comparing controls to STXBP1-RD iNeurons at group level (i.e., all control lines against all STXBP1-RD lines). Moreover, statistical tests comparing each STXBP1-RD line separately against the three control lines are reported. Correction for multiple testing was performed using the FDR method. Due to the explorative nature of this study, non-corrected p-values were used for interpretation of the data.

Suppl. Table 3: qPCR primers

Suppl. Table 4: Proteomics

Suppl. Table 5: Table with detailed information on healthy control iPSCs

Suppl. Fig. 1: Neuron & synapse morphology

A) Dendritic length was not different between controls and STXBP1-RD iNeuron lines. Sample sizes: 57-96/3-6 (n/N).

B) Dendritic complexity was similar between control and STXBP1-RD lines.

C) Right: Soma area was significantly smaller for D262V and R235X in patient-specific comparisons. 57-96/3-6 (n/N).

D) Density of presynaptic puncta, labelled by SYP immunostaining, was not different between controls and STXBP1-RD iNeuron lines. Sample sizes: 53-99/3-6 (n/N).

E) Density of postsynaptic puncta, labelled by PSD-95 immunostaining, was not different between controls and STXBP1-RD iNeuron lines. Sample sizes: 34-58/3-6 (n/N).

F) Intensity of SYP fluorescence was not different at group-level, but R235X iNeurons had a significantly lower staining intensity. Sample sizes: 51-99/3-6 (n/N).

G) Intensity of PSD-95 fluorescence did not differ at group-level between controls and STXBP1-RD. Sample sizes: 34-58/3-6 (n/N).

Data are presented in Tukey plots with dots representing individual neurons. ** = $p<0.01$; * = $p<0.05$, # = $p<0.1$. Statistical details are listed in Table S2.

Data on the left of panels D-G are presented in Tukey plots, where data outside of 1.5 times the interquartile range are plotted individually. Data in panels A and C, and on the right of panels D-G are presented in Tukey plots with dots representing individual neurons.

Suppl. Fig. 2: MUNC18-1 protein and RNA levels are reduced in STXBP1-RD iNeurons.

A) Left: Dendritic MUNC18-1 levels were significantly reduced in STXBP1-RD iNeurons compared to control iNeurons. Right: Significant reductions of MUNC18-1 levels were observed for D207G and S241fs iNeurons.

B) Left: Extra-dendritic (axonal) MUNC18-1 levels were significantly reduced in STXBP1-RD iNeurons compared to control iNeurons. Right: Significant reductions of MUNC18-1 levels were observed for D207G and S241fs iNeurons.

C) Schematic presentation of STXBP1 RNA primers for qPCR analysis. Variant sites are depicted in red. qPCR was performed on cell lysates with primers targeting the 5’, center and 3’ regions of MUNC18-1 mRNA.

D) RNA levels targeted by the ‘STXBP1 5’ primers were reduced in STXBP1-RD iNeurons compared to controls.

E) RNA levels targeted by the 'STXBP1 center' primers were reduced in STXBP1-RD iNeurons.

F) Patient-specific reduction in Syntaxin-1A RNA levels were observed for R235Q iNeurons.

G) No significant effects were found for Syntaxin-1B RNA levels.

H) SNAP25 RNA levels were not significantly different in patient-specific comparisons.

Data on the left of panels A, B, D and E are presented in Tukey plots, where data outside of 1.5 times the interquartile range are plotted individually. Data on the right of panels A and B are presented in Tukey plots with dots representing individual neurons, in D and E, and F-H individual data points are shown. ** = $p<0.01$; * = $p<0.05$, # = $p<0.1$. Statistical details are listed in Table S2. Sample sizes immunocytochemistry: 98-130/5 (n/N). Sample sizes western blot: 2-10 (N). Sample sizes qPCR: 5 (N).

Suppl. Fig. 3: Selection of evoked responses

A) A subset of iNeurons showed a synaptic response below the cut-off criterion of 400pA (red bar) and were excluded from the analysis of evoked synaptic transmission.

B) Histogram showing the distribution of the total deviation ratio (contribution of additional peaks in multi-peaked EPSC responses) for all EPSC responses in the dataset.

C) Visual inspection of EPSC responses was performed for a manual inclusion/exclusion decision. Individual dots represent individual neurons.

D) Histogram of the total deviation ratio as in B, but bars were split by user decision as in C. A cut-off criterion was applied at the indicated boundary, as this is the last TDR bin in which user include > user exclude.

Suppl. Fig. 4: Basal neurotransmission is unaltered in single STXBP1-RD iNeurons

A) No differences were observed in mEPSC amplitude, either at group-level (left) or patient-level comparisons. Sample sizes: 10-52/3-6 (n/N).

B) No differences were observed in mEPSC frequency, either at group-level (left) or patient-level comparisons. Sample sizes: 8-35/3-6 (n/N).

C) No changes at group-level (left) or patient-level comparisons in EPSC charge. Sample sizes: 26-62/3-6 (n/N).

Data on the left of panels A-C are presented in Tukey plots, where data outside of 1.5 times the interquartile range are plotted individually. Data on the right are presented in Tukey plots with dots representing individual neurons. ** = $p<0.01$; * = $p<0.05$, # = $p<0.1$. Statistical details are listed in Table S2.

Suppl. Fig. 5: No overarching changes in short-term plasticity of STXBP1-RD iNeurons

A) Left: Typical example of a paired-pulse recording, showing two EPSC responses to two action potentials delivered with 50ms inter-pulse interval. Paired-pulse ratio is calculated as the amplitude of the second peak over the first peak. Right: Typical example of a brief train stimulation (5 action potentials at 5 Hz) followed by a single stimulation 2 seconds after the end of the train. Synaptic depression is quantified as the amplitude of the last response in the train over the first response. Recovery after depression is quantified as the amplitude of the response 2 seconds after the train over the first response of the train.

B) No group-level differences were observed between patient and control lines in synaptic depression in response to a 10Hz train. However, iNeurons with R235X variant showed a significantly higher synaptic depression ratio compared to controls. Sample sizes: 25-56/3-6 (n/N).

C) No group-level differences were observed between patient and control lines in synaptic depression in response to a 20Hz train. However, iNeurons with R235X variant showed a significantly higher synaptic depression ratio compared to controls. Sample sizes: 23-48/3-6 (n/N).

D) No group-level or patient-specific differences were found in the response 2 seconds after synaptic depression induced by a 5Hz stimulation train. Sample sizes: 22-54/3-6 (n/N).

E) No group-level or patient-specific differences were found in the response 2 seconds after synaptic depression induced by a 10Hz stimulation train. Sample sizes: 25-57/3-6 (n/N).

F) No group-level or patient-specific differences were found in the response 2 seconds after synaptic depression induced by a 20Hz stimulation train. Sample sizes: 24-49/3-6 (n/N).

G) Typical example of the synaptic response to a sustained high-frequency (80 action potentials at 40Hz) stimulation train, followed by two single pulses to assess pool recovery 2 seconds and 60 seconds after the train.

H) No group-level or patient-specific differences were found in the total amount of charge transferred during the high-frequency train stimulation. Sample sizes: 22-47/3-6 (n/N).

I) Left: No group-level differences were found in RRP pool size. Right: Intronic iNeurons showed a significant increase in the estimated size of the RRP compared to controls. Sample sizes: 19-42/3-6 (n/N).

J) No group-level or patient-specific differences were found in recovery at 2 seconds post-high frequency stimulation. Sample sizes: 24-46/3-6 (n/N).

K) No group-level or patient-specific differences were found in recovery at 60 seconds post-high frequency stimulation. Sample sizes: 22-46/3-6 (n/N).

Data on the left of panels B-F, and H-K are presented in Tukey plots, where data outside of 1.5 times the interquartile range are plotted individually. Data on the right are presented in Tukey plots with dots representing individual neurons. ** = $p<0.01$; * = $p<0.05$, # = $p<0.1$. Statistical details are listed in Table S2.

Suppl. Fig. 6: Activity is affected in STXBP1-RD iNeuron networks

A) Group-level baseline burst frequency was not different between control and STXBP1-RD iNetworks.

B) Left: Baseline interburst interval (IBI) was not affected in group-level comparison of STXBP1-RD iNetworks. Right: Patient-level decreases were observed for D207G, R235X, S241fs iNetworks, and significant increase for Intronic iNetworks.

C) Group-level baseline CoV of IBIs was increased in STXBP1-RD iNetworks.

D) No group-level or patient-specific effects were found in mean burst amplitude at baseline.

E) Left: Baseline mean event area was decreased in group-level comparison between STXBP1-RD and control iNetworks. Right: No effects were found in patient-specific comparisons.

F) Left: Baseline rise time (i.e. time to reach peak amplitude after start of event) was significantly shorter in STXBP1-RD iNeuron networks. Right: No effects were found in patient-specific comparisons.

G) No significant effects were observed in baseline decay time (i.e. time from peak amplitude to end of event).

H) Left: Baseline event duration was shorter in STXBP1-RD iNeuron networks. Right: No significant effects were observed in patient-specific comparisons.

I) Burst frequency after 4-AP application was significantly increased in STXBP1-RD iNetworks.

J) Left: 4-AP IBI was significantly smaller in STBXP1-RD iNetworks. Right: IBIs were significantly shorter in R235Q and Intronic iNetworks.

K) Interburst interval CoV after 4-AP application was significantly higher in STXBP1-RD iNetworks.

L) Mean burst amplitude after 4-AP application was not different in STXBP1-RD iNeuron networks in group-level and patient-level comparisons.

M) Left: 4-AP mean event area was significantly smaller in STXBP1-RD iNetworks. Right: No significant effects were found in patient-level comparisons.

N) Left: Rise time after 4-AP application was significantly shorter in STXBP1-RD iNeuron networks. Right: No significant effects were observed in patient-level comparisons.

O) Left: STXBP1-RD iNetworks presented significantly smaller 4-AP decay time. Right: No significant effects were observed in patient-level comparisons.

P) Left: Burst duration after 4-AP application was shorter in STXBP1-RD iNeuron networks. Right: No significant effects were observed in patient-level comparisons.

Data in panels A and I, and on the left of panels B-H and J-P are presented in Tukey plots, where data outside of 1.5 times the interquartile range are plotted individually. Data on the right are presented in Tukey plots with dots representing individual networks. ** = $p<0.01$; * = $p<0.05$, # = $p<0.1$. Statistical details are listed in Table S2. Sample size baseline: 16-19/5 (n/N). Sample size 4AP: 12-18/5 (n/N).

Suppl. Fig. 7: Reduced synchronicity in STXBP1-RD iNetworks

A) Baseline mean participation was not significantly different in STXBP1-RD iNetworks.
B) Top: group-level reduction in the fraction of neurons fully participating in network events in STXBP1-RD compared to control iNetworks. Bottom: Significant reductions were observed for D207G and R235Q iNetworks.
C) CoV values of participation were significantly increased in STXBP1-RD iNetworks.
D) After 4AP application, mean neuron participation was significantly reduced in STXBP1-RD iNetworks.
E) STXBP1-RD iNetworks showed significant reduction in fraction of neurons fully participating in network events.
F) 4AP CoV participation was increased in STXBP1-RD iNetworks.

Data in panels A, B top and C-F are presented in Tukey plots, where data outside of 1.5 times the interquartile range are plotted individually. Data in panel B bottom are presented in Tukey plots with dots representing individual networks. ** = $p<0.01$; * = $p<0.05$, # = $p<0.1$. Statistical details are listed in Table S2. Sample size baseline: 16-19/5 (n/N). Sample size 4AP: 12-18/5 (n/N).

Suppl. Fig. 8: Coefficients of LDA network parameters

A) Coefficients of Linear Discriminant 1 between control and STXBP1-RD iNeuron networks (Fig. 7P). Network parameters are ranked based on weight.
B) Coefficients of Linear Discriminant 1 (left, accounting for 52% of model) and 2 (right, accounting for 25% of model) between all iNeuron network lines (Fig. 7Q). Network parameters are ranked based on weight.

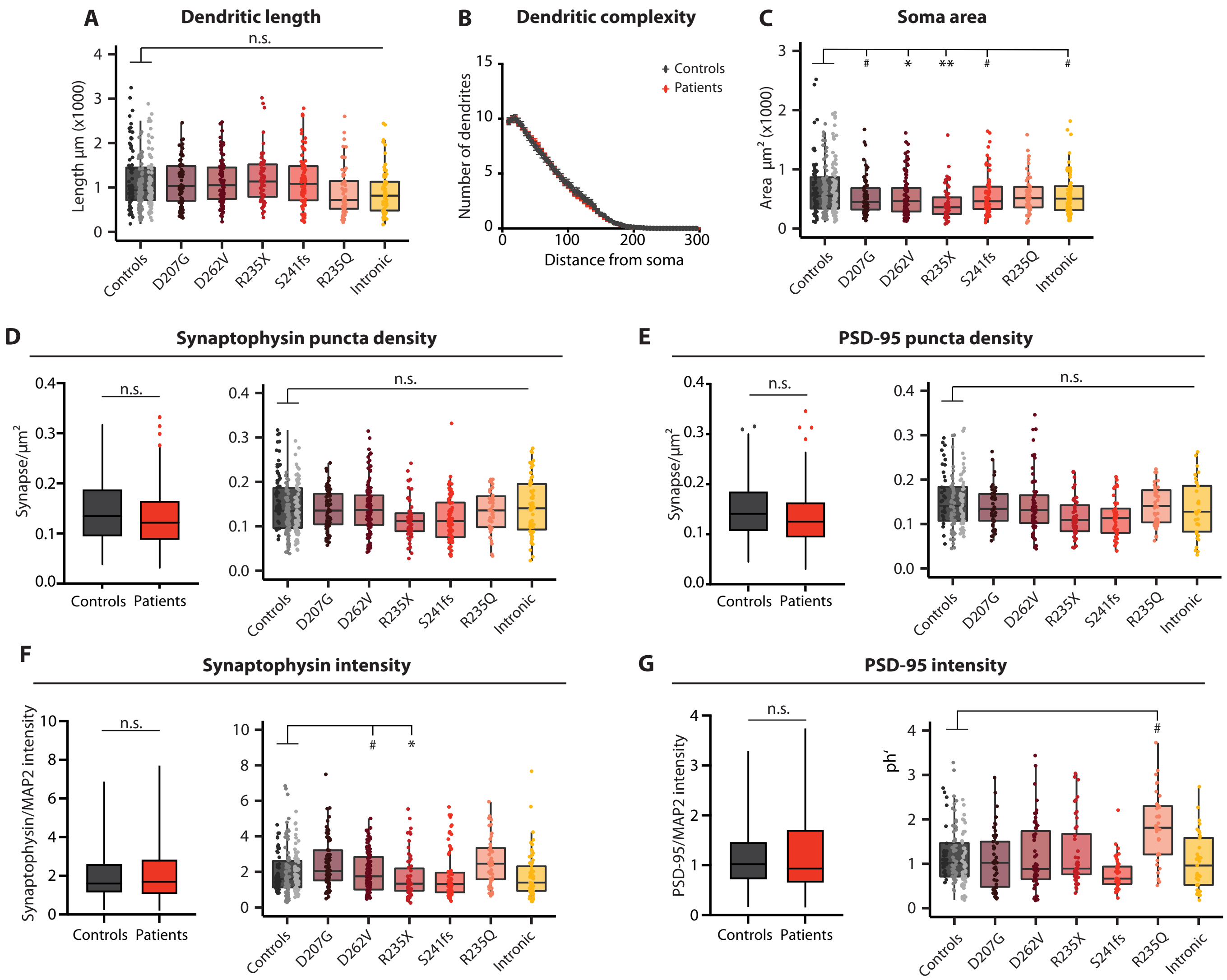

A
Dendritic length
n.s.
Length µm (x1000)
Controls
D207G
D262V
R235X
S241fs
R235Q
Intronic
B
Dendritic complexity
Controls
Patients
Number of dendrites
Distance from soma
C
Soma area
Area µm² (x1000)
D
Synaptophysin puncta density
Synapse/µm²
Patients
E
PSD-95 puncta density
F
Synaptophysin intensity
Synaptophysin/MAP2 intensity
G
PSD-95 intensity
PSD-95/MAP2 intensity

Figure S1

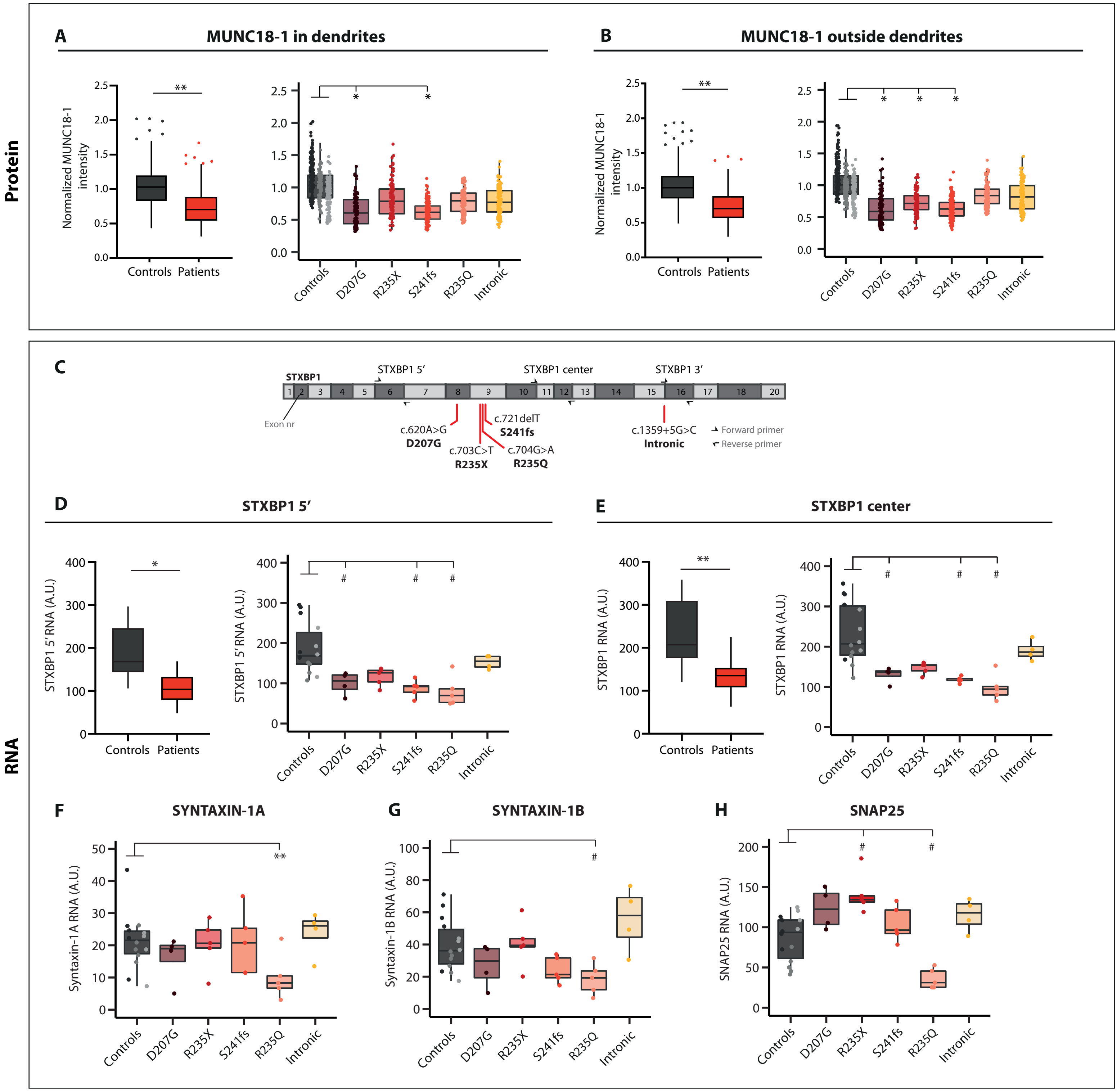

Protein
A
MUNC18-1 in dendrites
Normalized MUNC18-1 intensity
Controls
Patients
D207G
R235X
S241fs
R235Q
Intronic
B
MUNC18-1 outside dendrites
RNA
C
STXBP1
STXBP1 5′
STXBP1 center
STXBP1 3′
Exon nr
c.620A>G
D207G
c.721delT
S241fs
c.703C>T
R235X
c.704G>A
R235Q
c.1359+5G>C
Intronic
Forward primer
Reverse primer
D
STXBP1 5′
STXBP1 5′ RNA (A.U.)
E
STXBP1 center
STXBP1 RNA (A.U.)
F
SYNTAXIN-1A
Syntaxin-1A RNA (A.U.)
G
SYNTAXIN-1B
Syntaxin-1B RNA (A.U.)
H
SNAP25
SNAP25 RNA (A.U.)

Figure S2

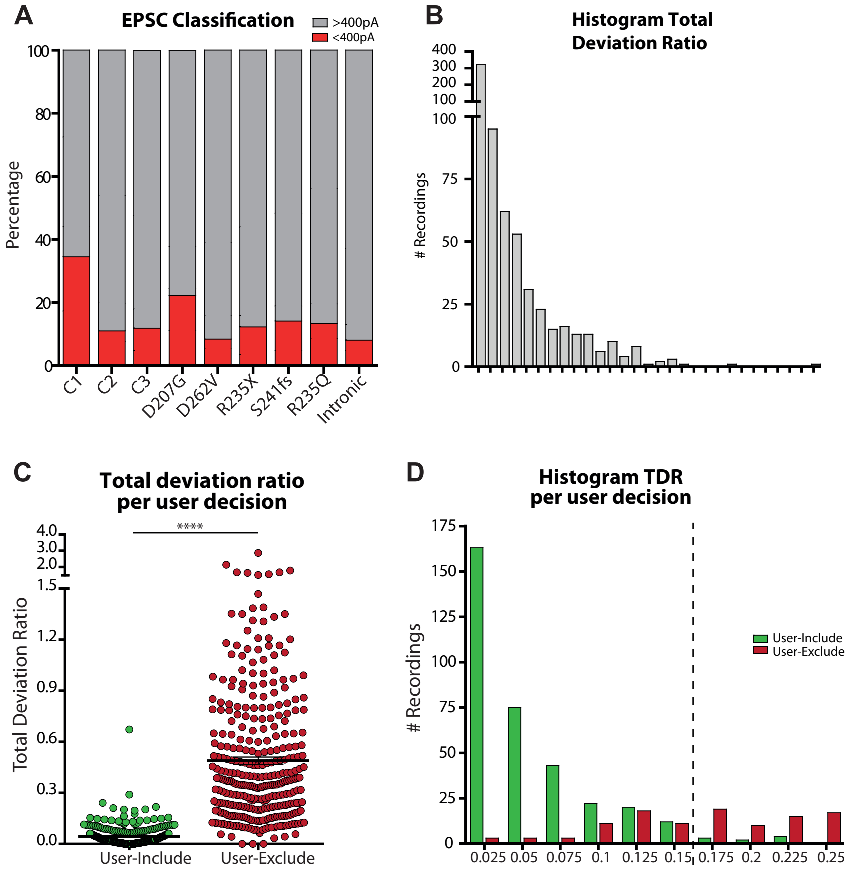
A
EPSC Classification
>400pA
<400pA
Percentage
C1
C2
C3
D207G
D262V
R235X
S241fs
R235Q
Intronic
B
Histogram Total
Deviation Ratio
Recordings
C
Total deviation ratio
per user decision
Total Deviation Ratio
****
User-Include
User-Exclude
D
Histogram TDR
per user decision
Recordings
User-Include
User-Exclude

Figure S3

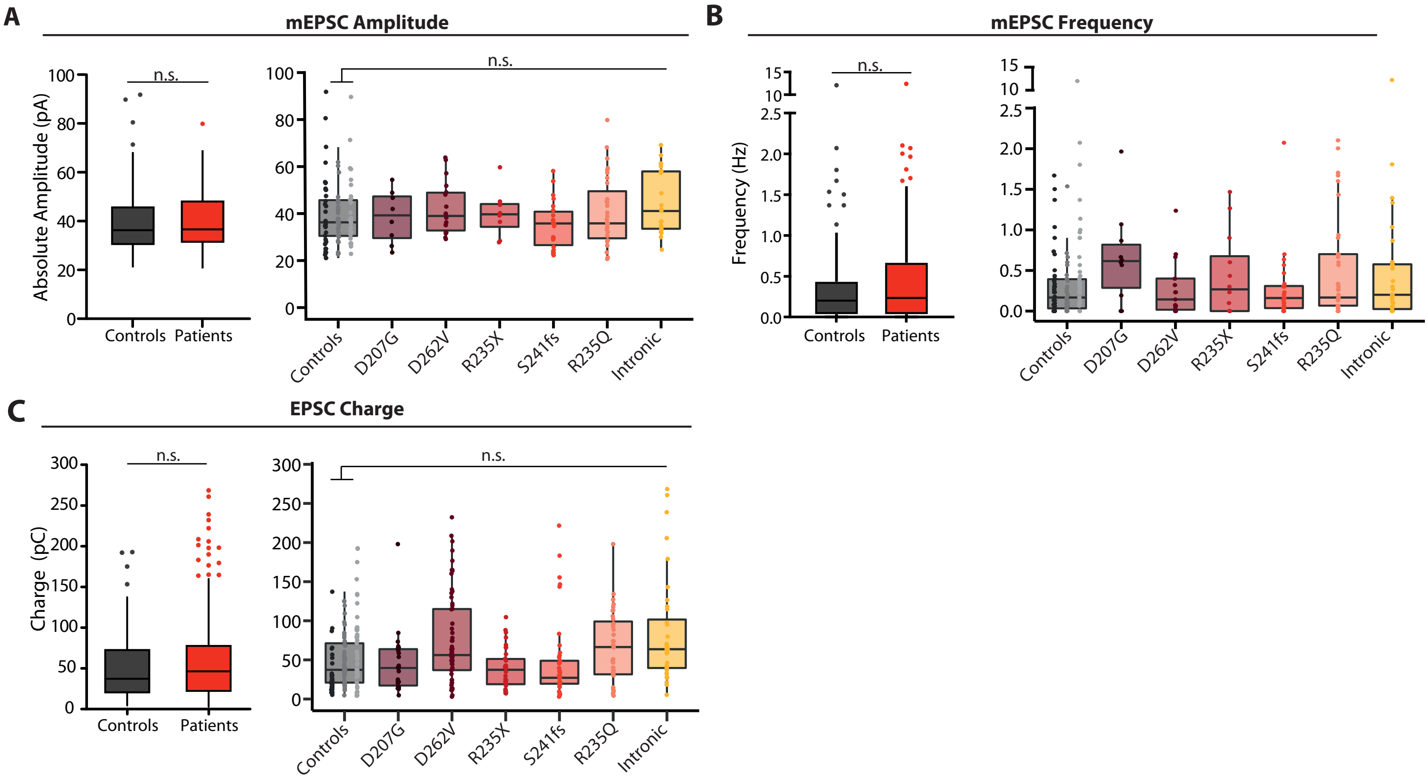
A
mEPSC Amplitude
Absolute Amplitude (pA)
n.s.
Controls
Patients
Controls
D207G
D262V
R235X
S241fs
R235Q
Intronic
B
mEPSC Frequency
Frequency (Hz)
C
EPSC Charge
Charge (pC)

Figure S4

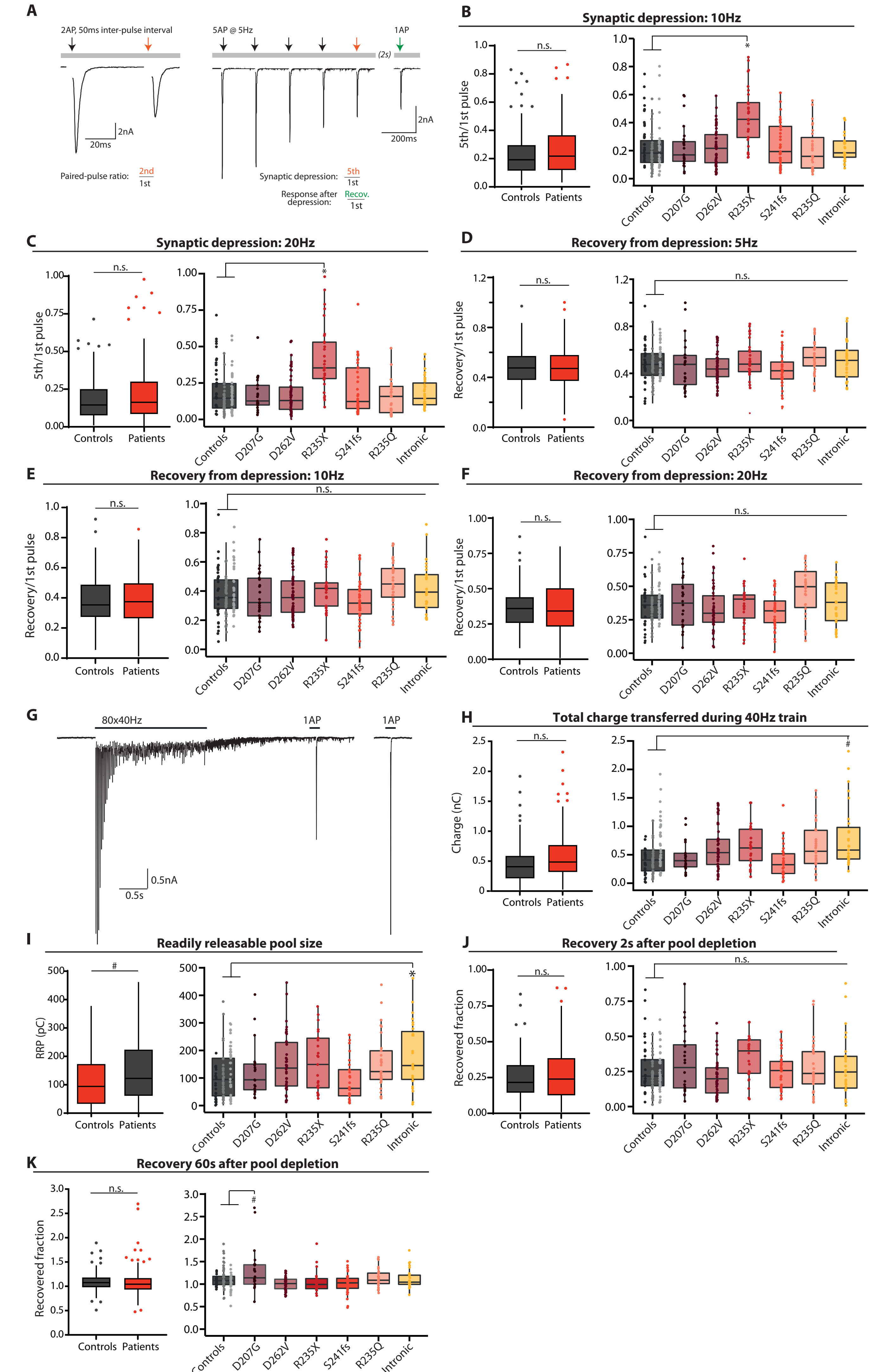

A
2AP, 50ms inter-pulse interval
2nA
20ms
Paired-pulse ratio: 2nd / 1st
5AP @ 5Hz
(2s)
1AP
2nA
200ms
Synaptic depression: 5th / 1st
Response after depression: Recov. / 1st
B
Synaptic depression: 10Hz
5th/1st pulse
n.s.
Controls
Patients
Controls
D207G
D262V
R235X
S241fs
R235Q
Intronic
C
Synaptic depression: 20Hz
D
Recovery from depression: 5Hz
Recovery/1st pulse
E
Recovery from depression: 10Hz
F
Recovery from depression: 20Hz
G
80x40Hz
1AP
0.5nA
0.5s
H
Total charge transferred during 40Hz train
Charge (nC)
I
Readily releasable pool size
RRP (pC)
J
Recovery 2s after pool depletion
Recovered fraction
K
Recovery 60s after pool depletion

Figure S5

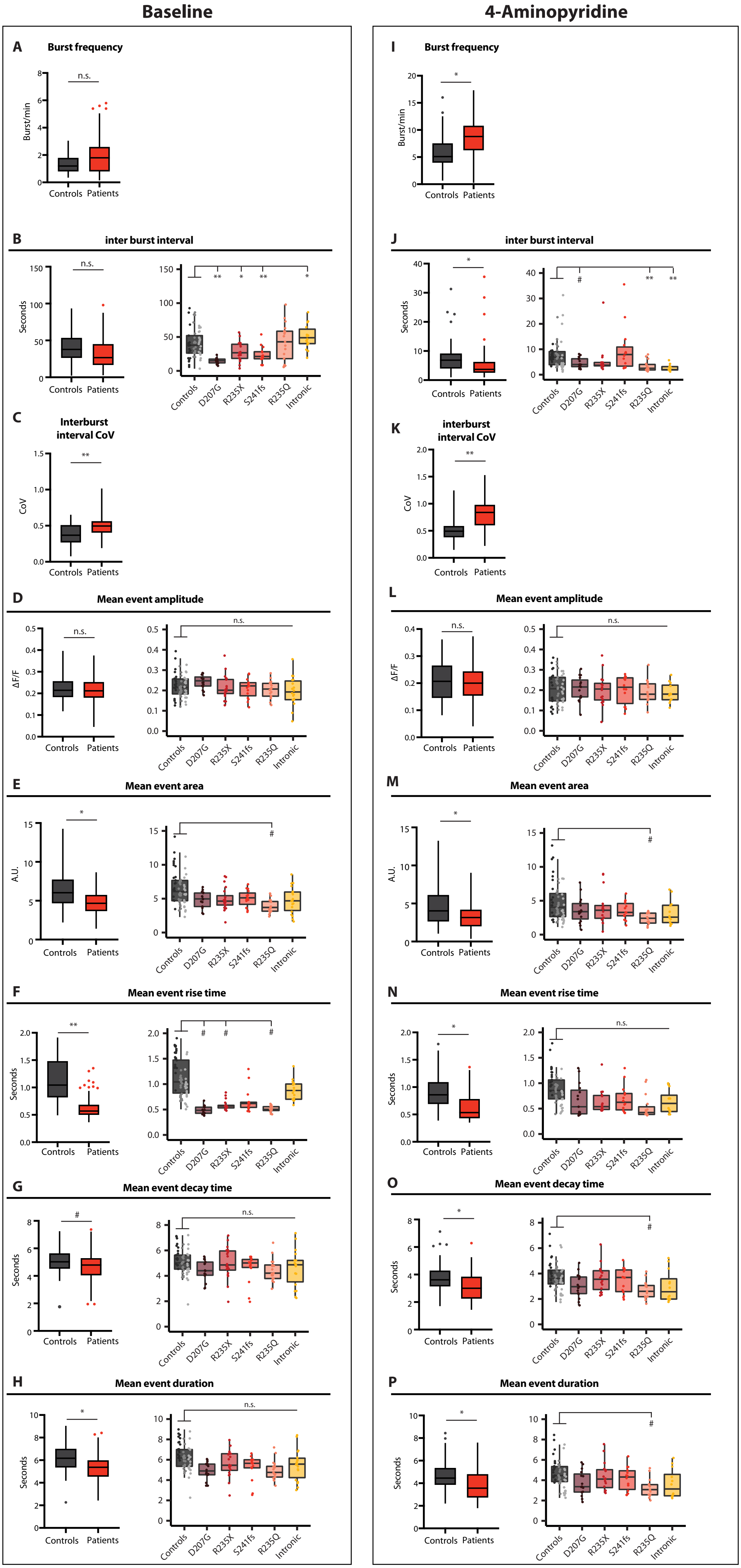
Baseline
4-Aminopyridine
A Burst frequency
Burst/min
n.s.
Controls Patients
I Burst frequency
B inter burst interval
Seconds
Controls D207G R235X S241fs R235Q Intronic
J inter burst interval
C Interburst interval CoV
CoV
K interburst interval CoV
D Mean event amplitude
ΔF/F
L Mean event amplitude
E Mean event area
A.U.
M Mean event area
F Mean event rise time
N Mean event rise time
G Mean event decay time
O Mean event decay time
H Mean event duration
P Mean event duration

Figure S6

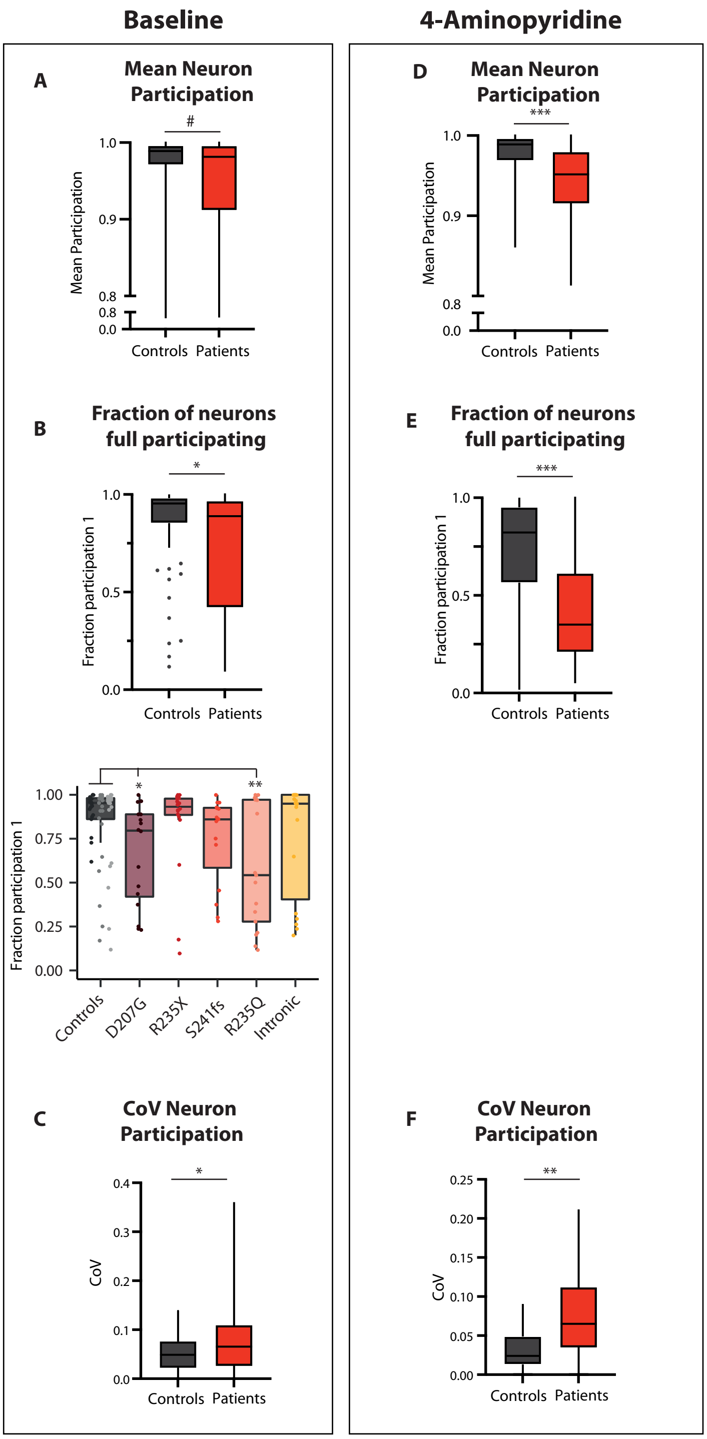
Baseline
4-Aminopyridine
A
Mean Neuron Participation
#
Mean Participation
1.0
0.9
0.8
0.8
0.0
Controls Patients
D
Mean Neuron Participation
***
Mean Participation
1.0
0.9
0.8
0.0
Controls Patients
B
Fraction of neurons full participating
*
Fraction participation 1
1.0
0.5
0.0
Controls Patients
E
Fraction of neurons full participating
***
Fraction participation 1
1.0
0.5
0.0
Controls Patients
Fraction participation 1
1.00
0.75
0.50
0.25
0.00
*
**
Controls
D207G
R235X
S241fs
R235Q
Intronic
C
CoV Neuron Participation
*
CoV
0.4
0.3
0.2
0.1
0.0
Controls Patients
F
CoV Neuron Participation
**
CoV
0.25
0.20
0.15
0.10
0.05
0.00
Controls Patients

Figure S7

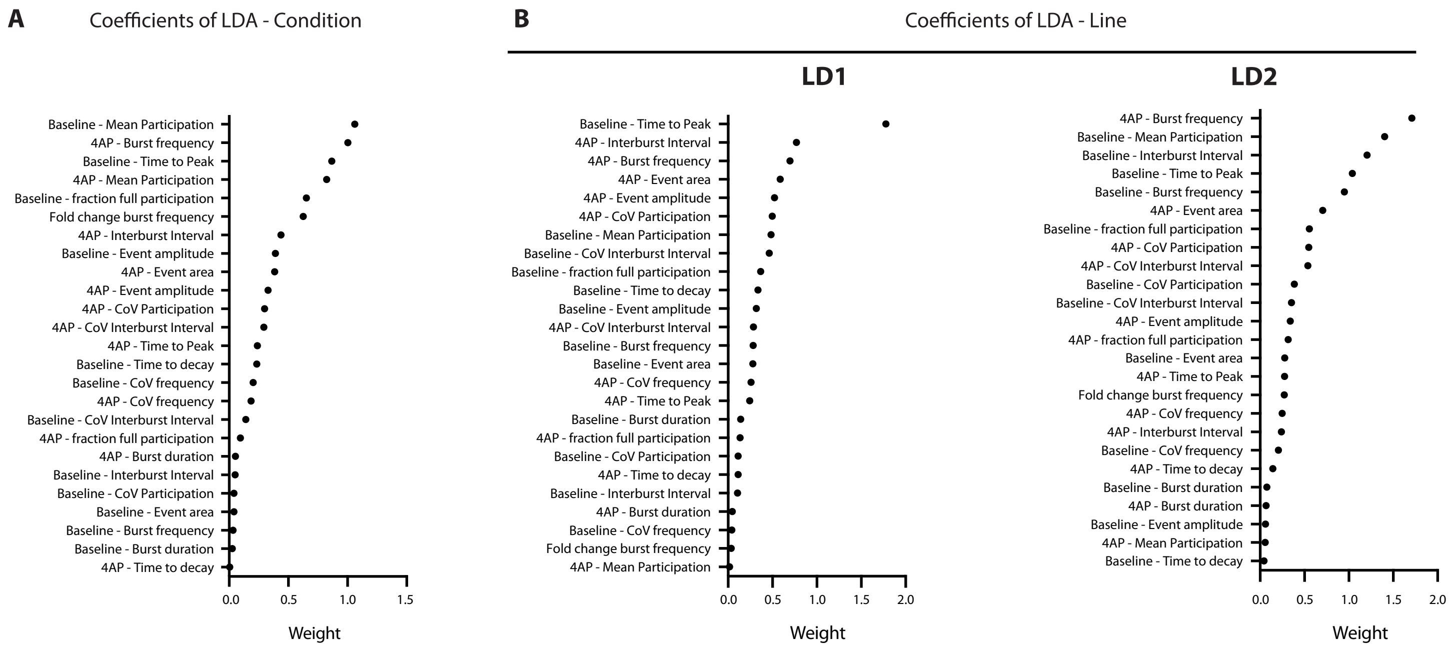
A
Coefficients of LDA - Condition
Baseline - Mean Participation
4AP - Burst frequency
Baseline - Time to Peak
4AP - Mean Participation
Baseline - fraction full participation
Fold change burst frequency
4AP - Interburst Interval
Baseline - Event amplitude
4AP - Event area
4AP - Event amplitude
4AP - CoV Participation
4AP - CoV Interburst Interval
4AP - Time to Peak
Baseline - Time to decay
Baseline - CoV frequency
4AP - CoV frequency
Baseline - CoV Interburst Interval
4AP - fraction full participation
4AP - Burst duration
Baseline - Interburst Interval
Baseline - CoV Participation
Baseline - Event area
Baseline - Burst frequency
Baseline - Burst duration
4AP - Time to decay
0.0
0.5
1.0
1.5
Weight
B
Coefficients of LDA - Line
LD1
Baseline - Time to Peak
4AP - Interburst Interval
4AP - Burst frequency
4AP - Event area
4AP - Event amplitude
4AP - CoV Participation
Baseline - Mean Participation
Baseline - CoV Interburst Interval
Baseline - fraction full participation
Baseline - Time to decay
Baseline - Event amplitude
4AP - CoV Interburst Interval
Baseline - Burst frequency
Baseline - Event area
4AP - CoV frequency
4AP - Time to Peak
Baseline - Burst duration
4AP - fraction full participation
Baseline - CoV Participation
4AP - Time to decay
Baseline - Interburst Interval
4AP - Burst duration
Baseline - CoV frequency
Fold change burst frequency
4AP - Mean Participation
0.0
0.5
1.0
1.5
2.0
Weight
LD2
4AP - Burst frequency
Baseline - Mean Participation
Baseline - Interburst Interval
Baseline - Time to Peak
Baseline - Burst frequency
4AP - Event area
Baseline - fraction full participation
4AP - CoV Participation
4AP - CoV Interburst Interval
Baseline - CoV Participation
Baseline - CoV Interburst Interval
4AP - Event amplitude
4AP - fraction full participation
Baseline - Event area
4AP - Time to Peak
Fold change burst frequency
4AP - CoV frequency
4AP - Interburst Interval
Baseline - CoV frequency
4AP - Time to decay
Baseline - Burst duration
4AP - Burst duration
Baseline - Event amplitude
4AP - Mean Participation
Baseline - Time to decay
0.0
0.5
1.0
1.5
2.0
Weight

Figure S8

Table S1: Table with clinical information

	P1 – D207G	P2 - SD262V	P3 – R235X	P4 – S241fs	P5 – R235Q[1]	P6 - Intronic
Mutation	c. 620 A>G;<br>p. Asp207Gly	c. 785 A>T<br>p. Asp262Val	c. 703 C>T;<br>p. Arg235*	c. 721delT;<br>p. Ser241fs	c. 704 G>A<br>p. Arg235Gln	c. 1359+5 G>C
Age at inclusion	6y3mo	20y	33y10m	3y6m	2y9m	17y5m
Sex	m	m	f	m	m	F
Developmental delay	Yes	Yes	Yes	Yes	Yes	Yes
Onset/Reason for referral to genetic testing	Developmental/psychomotor delay (age: 18 months)	Epileptic seizures (age: 1 week)	Absences (age: 2 months)	Convulsions, hypotonia (age: 4 months)	Generalized clonic seizures (age: 1 month)	Clustered tonic seizures, lack of eye contact (age: 4 weeks)
Communication	Multiple-word sentences	Unable to speak	Unable to speak; can produce sounds	Unable to speak; can produce sounds, non-verbal communication	Some sounds or words: delayed, poor babbling and comprehension	Unable to speak; can produce sounds
Epileptic seizures	No	Yes	Yes	Yes	Yes	Yes
Type of seizures	n/a		Focal seizures, followed by infactile spasms; tonic-clonic seizures; absences	Focal seizures with impaired awareness	Clonic	Focal seizures, tonic-clonic seizures, myoclonic seizures, atonic seizures
Seizures controlled?	n/a	No	No	Yes	Yes	No
AEDs used at time of inclusion	n/a	clobazam, nitrazepam; vigabatrin; lacosamide; midazolam (emergency medication)	levetiracetam, topiramate, lamotrigine; diazepam (emergency medication)	levetiracetam	levetiracetam	sodium valproate, lamotrigine
Psychiatric and behavioural features	Stereotypic behaviours; hyperactivity; hyperphagia; fulfils criteria for autism spectrum disorder. Insatiable.		Stereotypic behaviours; restlessness; anxiety	Stereotypic behaviours; restlessness		Stereotypic behaviours (including bruxism and self-injurious behaviour)
Neurodevelopmental features at inclusion	Able to walk unsupported.	Able to sit unsupported, but unable to walk.	Unable to sit unsupported. Severe spastic tetraparesis. Impaired pain sensation.	Able to sit unsupported and crawl. Ataxia. Hypotonia.	Able to walk unsupported, but unstable and broad based. Ataxia.	Able to walk few steps but unstable. Ataxia. Dystonia. Hypertonia. Impaired pain sensation. Oculomotor movement disorder.

[1]Case report published: Dilena et al. (2016)

Table S2: Table with statistical parameters

PARAMETER	LINE	SAMPLE SIZE (N/n)	ANOVA BETWEEN MODELS	SIGN. AFTER FDR?
**SYNAPSE PHYSIOLOGY – PATCH-CLAMP AUTAPSE PHYSIOLOGY**				
**MEPSC FREQUENCY**	Controls *vs* Patients		$\chi^2(1) = 0.152$, p=0.697	
	C1	52		
	C2	41		
	C3	33		
	P1	10	$\chi^2(1) = 0.029$, p=0.865	n.s.
	P2	23	$\chi^2(1) = 1.957$, p=0.162	n.s.
	P3	12	$\chi^2(1) = 0.618$, p=0.432	n.s.
	P4	24	$\chi^2(1) = 0.757$, p=0.384	n.s.
	P5	38	$\chi^2(1) = 0.279$, p=0.597	n.s.
	P6	29	$\chi^2(1) = 0.055$, p=0.815	n.s.
**MEPSC AMPLITUDE**	Controls *vs* Patients		$\chi^2(1) = 0.0764$, p=0.782	
	C1	35		
	C2	35		
	C3	30		
	P1	8	$\chi^2(1) = 0.495$, p=0.482	n.s.
	P2	18	$\chi^2(1) = 0.403$, p=0.526	n.s.
	P3	8	$\chi^2(1) = 0.522$, p=0.47	n.s.
	P4	22	$\chi^2(1) = 2.354$, p=0.125	n.s.
	P5	31	$\chi^2(1) = 0.212$, p=0.646	n.s.
	P6	23	$\chi^2(1) = 0.807$, p=0.369	n.s.
**PEAK EPSC AMPLITUDE**	Controls *vs* Patients		$\chi^2(1) = 0.67$, p=0.414	
	C1	37		
	C2	48		
	C3	46		
	P1	26	$\chi^2(1) = 0.16$, p=0.693	n.s.
	P2	61	$\chi^2(1) = 3.02$, p=0.082 #	n.s.
	P3	34	$\chi^2(1) = 0.12$ p=0.725	n.s.
	P4	41	$\chi^2(1) = 1.22$, p=0.27	n.s.
	P5	36	$\chi^2(1) = 1.25$, p=0.263	n.s.
	P6	36	$\chi^2(1) = 5.69$, p=0.017*	n.s.
**EPSC CHARGE**	Controls *vs* Patients		$\chi^2(1) = 0.848$, p=0.357	
	C1	36		
	C2	48		
	C3	45		
	P1	26	$\chi^2(1) = 0.027$, p=0.870	n.s.
	P2	62	$\chi^2(1) = 2.82$, p=0.093	n.s.
	P3	34	$\chi^2(1) = 0.33$, p=0.564	n.s.
	P4	41	$\chi^2(1) = 0.26$, p=0.608	n.s.
	P5	35	$\chi^2(1) = 1.26$, p=0.261	n.s.
	P6	36	$\chi^2(1) = 2.96$, p=0.085	n.s.
**PAIRED PULSE RATIO**	Controls *vs* Patients		$\chi^2(1)$ =0.6, p=0.439	
	C1	32		
	C2	43		
	C3	44		
	P1	27	$\chi^2(1)$ =0.099, p=0.753	n.s.
	P2	57	$\chi^2(1) = 0.0013$, p=0.971	n.s.
	P3	33	$\chi^2(1) = 4.492$, p=0.034*	n.s.
	P4	39	$\chi^2(1) = 0.278$, p=0.598	n.s.
	P5	37	$\chi^2(1) = 0.248$, p=0.619	n.s.
	P6	33	$\chi^2(1) = 0.399$, p=0.528	n.s.
**5HZ SYNAPTIC DEPRESSION**	Controls *vs* Patients		$\chi^2(1) = 0.869$, p=0.351	
	C1	31		
	C2	41		
	C3	39		
	P1	21	$\chi^2(1) = 0.096$ p=0.756	n.s.
	P2	55	$\chi^2(1) = 0.0071$, p=0.933	n.s.
	P3	32	$\chi^2(1) = 5.329$, p=0.021*	n.s.
	P4	37	$\chi^2(1) = 0.315$, p=0.575	n.s.
	P5	35	$\chi^2(1) = 0.020$, p=0.886	n.s.
	P6	28	$\chi^2(1) = 0.332$, p=0.564	n.s.
**5HZ RECOVERY AFTER DEPRESSION**	Controls *vs* Patients		$\chi^2(1) = 0.0073$, p=0.932	
	C1	32		
	C2	41		
	C3	39		
	P1	22	$\chi^2(1) = 0.053$, p=0.828	n.s.
	P2	54	$\chi^2(1) = 1.056$, p=0.304	n.s.
	P3	32	$\chi^2(1) = 0.018$, p=0.893	n.s.
	P4	38	$\chi^2(1) = 1.577$, p=0.209	n.s.
	P5	34	$\chi^2(1) = 0.867$, p=0.352	n.s.
	P6	29	$\chi^2(1) = 0.891$, p=0.345	n.s.
**10HZ SYNAPTIC DEPRESSION**	Controls *vs* Patients		$\chi^2(1) = 0.264$, p=0.608	
	C1	29		
	C2	40		

Table S2: Table with statistical parameters

	C3	35		
	P1	25	$\chi^2(1) = 0.023$, p=0.880	n.s.
	P2	56	$\chi^2(1) = 0.013$, p=0.911	n.s.
	P3	29	$\chi^2(1) = 5.357$, p=0.021*	n.s.
	P4	37	$\chi^2(1) = 0.169$, p=0.681	n.s.
	P5	31	$\chi^2(1) = 0.475$, p=0.491	n.s.
	P6	28	$\chi^2(1) = 0.038$, p=0.845	n.s.
**10HZ RECOVERY FROM DEPRESSION**	Controls *vs* Patients		$\chi^2(1) = 0.175$, p=0.676	
	C1	30		
	C2	40		
	C3	35		
	P1	25	$\chi^2(1) = 0.246$, p=0.620	n.s.
	P2	57	$\chi^2(1) = 0.026$, p=0.872	n.s.
	P3	29	$\chi^2(1) = 0.213$, p=0.644	n.s.
	P4	37	$\chi^2(1) = 0.876$, p=0.349	n.s.
	P5	31	$\chi^2(1) = 1.832$, p=0.176	n.s.
	P6	29	$\chi^2(1) = 1.56$, p=0.206	n.s.
**20HZ SYNAPTIC DEPRESSION**	Controls *vs* Patients		$\chi^2(1) = 0.237$, p=0.627	
	C1	29		
	C2	38		
	C3	37		
	P1	23	$\chi^2(1) = 0.026$, p=0.873	n.s.
	P2	48	$\chi^2(1) = 0.012$, p=0.912	n.s.
	P3	30	$\chi^2(1) = 5.32$, p=0.021*	n.s.
	P4	36	$\chi^2(1) = 0.273$, p=0.542	n.s.
	P5	29	$\chi^2(1) = 0.700$, p=0.403	n.s.
	P6	31	$\chi^2(1) = 2*10^{-4}$, p=0.99	n.s.
**20HZ RECOVERY FROM DEPRESSION**	Controls *vs* Patients		$\chi^2(1) = 0$, p=0.999	
	C1	30		
	C2	38		
	C3	38		
	P1	24	$\chi^2(1) = 0.037$, p=0.847	n.s.
	P2	49	$\chi^2(1) = 0.034$, p=0.854	n.s.
	P3	30	$\chi^2(1) = 1.729$, p=0.189	n.s.
	P4	36	$\chi^2(1) = 1.561$, p=0.212	n.s.
	P5	30	$\chi^2(1) = 2.447$, p=0.118	n.s.
	P6	32	$\chi^2(1) = 0.142$, p=0.707	n.s.
**40HZ RECOVERY AFTER 2 SECONDS**	Controls *vs* Patients		$\chi^2(1) = 0.463$, p=0.496	
	C1	27		
	C2	37		
	C3	38		
	P1	24	$\chi^2(1) = 0.905$, p=0.341	n.s.
	P2	46	$\chi^2(1) = 0.778$, p=0.378	n.s.
	P3	24	$\chi^2(1) = 2.010$, p=0.156	n.s.
	P4	32	$\chi^2(1) = 6*10^{-4}$, p=0.980	n.s.
	P5	29	$\chi^2(1) = 0.183$, p=0.668	n.s.
	P6	27	$\chi^2(1) = 0.690$, p=0.406	n.s.
**40HZ RECOVERY AFTER 60 SECONDS**	Controls *vs* Patients		$\chi^2(1) = 0.021$, p=0.885	
	C1	27		
	C2	36		
	C3	30		
	P1	22	$\chi^2(1) = 3.47$, p=0.0626 #	n.s.
	P2	46	$\chi^2(1) = 0.883$, p=0.347	n.s.
	P3	24	$\chi^2(1) = 0.45$, p=0.704	n.s.
	P4	32	$\chi^2(1) = 0.973$, p=0.324	n.s.
	P5	27	$\chi^2(1) = 0.215$, p=0.643	n.s.
	P6	27	$\chi^2(1) = 0.014$, p=0.906	n.s.
**40HZ TOTAL CHARGE TRANSFERRED**	Controls *vs* Patients		$\chi^2(1) = 1.883$, p=0.17	
	C1	26		
	C2	36		
	C3	38		
	P1	22	$\chi^2(1) = 0.0412$, p=0.839	n.s.
	P2	47	$\chi^2(1) = 1.116$, p=0.291	n.s.
	P3	25	$\chi^2(1) = 1.009$, p=0.315	n.s.
	P4	31	$\chi^2(1) = 0.237$, p=0.626	n.s.
	P5	29	$\chi^2(1) = 0.879$, p=0.349	n.s.
	P6	27	$\chi^2(1) = 3.532$, p=0.060 #	n.s.
**RRP ESTIMATION**	Controls *vs* Patients		$\chi^2(1) = 3.767$, p=0.0523 #	
	C1	24		
	C2	31		
	C3	35		
	P1	19	$\chi^2(1) = 0.943$, p=0.332	n.s.
	P2	42	$\chi^2(1) = 2.427$, p=0.119	n.s.
	P3	25	$\chi^2(1) = 2.034$, p=0.154	n.s.
	P4	25	$\chi^2(1) = 0.149$, p=0.700	n.s.
	P5	29	$\chi^2(1) = 1.333$, p=0.248	n.s.

Table S2: Table with statistical parameters

	P6	27	$\chi^2(1) = 4.318$, p=0.0378*	n.s.
**DENDRITIC & SYNAPTIC MORPHOLOGY**				
**DENDRITE LENGTH**	Controls *vs* Patients		$\chi^2(1) = 0.172$, p=0.678	
	C1	67		
	C2	82		
	C3	87		
	P1	73	$\chi^2(1) = 139$, p=0.710	n.s.
	P2	96	$\chi^2(1) = 0.228$, p=0.633	n.s.
	P3	57	$\chi^2(1) = 0.013$, p=0.909	n.s.
	P4	70	$\chi^2(1) =0.207$, p=0.650	n.s.
	P5	57	$\chi^2(1) = 1.290$, p=0.256	n.s.
	P6	70	$\chi^2(1) = 0.846$, p=0.358	n.s.
**SOMA AREA**	Controls *vs* Patients		$\chi^2(1) = 9.191$, p=0.00243 **	
	C1	65		
	C2	82		
	C3	87		
	P1	71	$\chi^2(1) = 3.098$, p=0.078 #	n.s.
	P2	97	$\chi^2(1) = 3.99$, p=0.046 *	n.s.
	P3	56	$\chi^2(1) = 7.23$, p=0.0072 **	*
	P4	69	$\chi^2(1) = 3.53$, p=0.060 #	n.s.
	P5	56	$\chi^2(1) = 1.89$, p=0.169	n.s.
	P6	70	$\chi^2(1) = 2.75$, p=0.097 #	n.s.
**DENSITY PRE-SYNAPTIC PUNCTA**	Controls *vs* Patients		$\chi^2(1) = 0.993$, p=0.319	
	C1	53		
	C2	67		
	C3	77		
	P1	73	$\chi^2(1) = 0.076$, p=0.783	n.s.
	P2	99	$\chi^2(1) = 0.030$, p=0.863	n.s.
	P3	58	$\chi^2(1) = 0.424$, p=0.515	n.s.
	P4	71	$\chi^2(1) = 0.363$, p=0.547	n.s.
	P5	43	$\chi^2(1) = 0.375$, p=0.540	n.s.
	P6	63	$\chi^2(1) = 0$, p=0.999	n.s.
**SYNAPTOPHYSIN INTENSITY**	Controls *vs* Patients		$\chi^2(1) = 0.629$, p=0.428	
	C1	51		
	C2	67		
	C3	76		
	P1	73	$\chi^2(1) = 2.13$, p=0.144	n.s.
	P2	99	$\chi^2(1) = 2.84$, p=0.092 #	n.s.
	P3	58	$\chi^2(1) = 4.26$, p=0.039 *	n.s.
	P4	71	$\chi^2(1) = 0.25$, p=0.616	n.s.
	P5	43	$\chi^2(1) = 2.36$, p=0.125	n.s.
	P6	63	$\chi^2(1) = 0.98$, p=0.322	n.s.
**DENSITY POST-SYNAPTIC PUNCTA**	Controls *vs* Patients		$\chi^2(1) = 5.034$, p=0.0249 *	
	C1	34		
	C2	49		
	C3	53		
	P1	45	$\chi^2(1) = 1.045$, p=0.307	n.s.
	P2	58	$\chi^2(1) = 0.484$, p=0.486	n.s.
	P3	44	$\chi^2(1) = 0.449$, p=0.503	n.s.
	P4	41	$\chi^2(1) = 1.476$, p=0.224	n.s.
	P5	38	$\chi^2(1) = 1.142$, p=0.285	n.s.
	P6	33	$\chi^2(1) = 0.706$, p=0.401	n.s.
**INTENSITY POST-SYNAPTIC PUNCTA**	Controls *vs* Patients		$\chi^2(1) = 0.0081$, p=0.9283	
	C1	34		
	C2	48		
	C3	53		
	P1	45	$\chi^2(1) = 1.820$, p=0.177	n.s.
	P2	58	$\chi^2(1) = 1.159$, p=0.282	n.s.
	P3	44	$\chi^2(1) = 0.172$, p=0.678	n.s.
	P4	42	$\chi^2(1) = 0.723$, p=0.392	n.s.
	P5	37	$\chi^2(1) = 2.823$, p=0.093	n.s.
	P6	33	$\chi^2(1) = 2.00$, p=0.157	n.s.
**MUNC18-1/STXBP1 LEVELS - IMMUNOCYTOCHEMISTRY**				
**TOTAL MUNC18-1 INTENSITY**	Controls *vs* Patients		$\chi^2(1) = 8.607$, p=0.00335 **	
	C1	130		
	C2	99		
	C3	122		
	P1	107	$\chi^2(1) = 4.97$, p=0.0258 *	#
	P3	105	$\chi^2(1) = 2.903$, p=0.0884 #	n.s.
	P4	122	$\chi^2(1) = 5.133$, p=0.0235 *	#
	P5	107	$\chi^2(1) = 1.836$, p=0.175	n.s.
	P6	118	$\chi^2(1) = 2.284$, p=0.131	n.s.
**SYNAPTIC MUNC18-1 INTENSITY**	Controls *vs* Patients		$\chi^2(1) = 9.391$, p=0.00218 **	
	C1	128		
	C2	98		

Table S2: Table with statistical parameters

	C3	122		
	P1	107	$\chi^2(1) = 3.668$, p=0.0555 #	#
	P3	105	$\chi^2(1) = 3.531$, p= 0.060 #	#
	P4	122	$\chi^2(1) = 3.626$, p=0.057 #	#
	P5	107	$\chi^2(1) = 1.924$, p=0.165	n.s.
	P6	118	$\chi^2(1) = 2.281$, p=0.131	n.s.
**DENDRITIC MUNC18-1 INTENSITY**	Controls *vs* Patients		$\chi^2(1) = 8.951$, p=0.00278 **	
	C1	129		
	C2	99		
	C3	122		
	P1	107	$\chi^2(1) =4.596$, p=0.030*	#
	P3	105	$\chi^2(1) = 2.007$, p=0.157	n.s.
	P4	122	$\chi^2(1) = 4.932$, p=0.026*	#
	P5	107	$\chi^2(1) = 2.436$, p=0.118	n.s.
	P6	118	$\chi^2(1) = 2.655$, p=0.103	n.s.
**NON-DENDRITIC MUNC18-1 INTENSITY**	Controls *vs* Patients		$\chi^2(1) = 8.669$, p=0.00324 **	
	C1	130		
	C2	99		
	C3	121		
	P1	107	$\chi^2(1) = 4.924$, p=0.0265 *	#
	P3	105	$\chi^2(1) = 3.645$, p=0.056 #	#
	P4	122	$\chi^2(1) = 5.091$, p=0.0241 *	#
	P5	107	$\chi^2(1) = 1.670$, p=0.196	n.s.
	P6	118	$\chi^2(1) = 2.156$, p=0.142	n.s.
**WESTERN BLOT**				
**MUNC18-1/GAMMA-TUB**	Controls *vs* Patients		$\chi^2(1) = 7.5612$, p=0.0059 **	
**QPCR**				
**STXBP1**	Controls *vs* Patients		$\chi^2(1) = 6.940$, p=0.0084 **	
	C1	5		
	C2	5		
	C3	4		
	P1	4	$\chi^2(1) = 3.140$, p=0.0764 #	n.s.
	P3	5	$\chi^2(1) = 1.858$, p=0.173	n.s.
	P4	5	$\chi^2(1) = 2.932$, p=0.0868 #	n.s.
	P5	5	$\chi^2(1) = 3.683$, p=0.0550 #	n.s.
	P6	4	$\chi^2(1) = 1.00$, p=0.317	n.s.
**STXBP1 – 3'UTR**	Controls *vs* Patients		$\chi^2(1) = 11.86$, p=0.0005736 ***	
	C1	5		
	C2	5		
	C3	4		
	P1	4	$\chi^2(1) = 3.6655$, p=0.0555 #	#
	P3	5	$\chi^2(1) = 3.2866$, p=0.06985 #	#
	P4	5	$\chi^2(1) = 4.2151$, p=0.04007 *	#
	P5	5	$\chi^2(1) = 4.3465$, p=0.03709 *	#
	P6	4	$\chi^2(1) = 3.5033$, p=0.06125 #	#
**STXBP1 – 5'UTR**	Controls *vs* Patients		$\chi^2(1) = 6.6189$, p=0.01009 *	
	C1	5		
	C2	5		
	C3	4		
	P1	4	$\chi^2(1) = 3.010$, p=0.0827 #	n.s.
	P3	5	$\chi^2(1) = 1.878$, p=0.171	n.s.
	P4	5	$\chi^2(1) = 3.241$, p=0.0718 #	n.s.
	P5	5	$\chi^2(1) = 3.618$, p=0.0572 #	n.s.
	P6	4	$\chi^2(1) = 0.747$, p=0.388	n.s.
**STX1A**	Controls *vs* Patients		$\chi^2(1) = 0.6055$, p=0.4365	
	C1	5		
	C2	5		
	C3	4		
	P1	4	$\chi^2(1) = 0.9742$, p=0.3236	n.s.
	P3	5	$\chi^2(1) = 0.4328$, p=0.5106	n.s.
	P4	5	$\chi^2(1) = 0.2258$, p=0.6347	n.s.
	P5	5	$\chi^2(1) = 7.7948$, p=0.00524 **	*
	P6	4	$\chi^2(1) = 2.5067$, p=0.1134	n.s.
**STX1B**	Controls *vs* Patients		$\chi^2(1) = 0.444$, p=0.505	
	C1	5		
	C2	5		
	C3	4		
	P1	4	$\chi^2(1) = 1.117$, p=0.291	n.s.
	P3	5	$\chi^2(1) = 0.0075$, p=0.931	n.s.
	P4	5	$\chi^2(1) = 1.978$, p=0.160	n.s.
	P5	5	$\chi^2(1) = 3.151$, p=0.076	n.s.
	P6	4	$\chi^2(1) = 2.241$, p=0.134	n.s.
**SNAP25**	Controls *vs* Patients		$\chi^2(1) = 0.465$, p=0.495	
	C1	5		
	C2	5		
	C3	4		
	P1	4	$\chi^2(1) = 1.612$, p=0.204	n.s.
	P3	5	$\chi^2(1) = 3.409$, p=0.065	n.s.

Table S2: Table with statistical parameters

	P4	5	$\chi^2(1)$ = 0.488, p=0.485	n.s.
	P5	5	$\chi^2(1)$ = 2.931, p=0.087	n.s.
	P6	4	$\chi^2(1)$ = 1.014, p=0.314	n.s.
**NETWORK ACTIVITY – CALCIUM IMAGING**				
**BASELINE - FREQUENCY**	Controls *vs* Patients		$\chi^2(1)$ = 1.6659, p=0.197	
	C1	19		
	C2	19		
	C3	19		
	P1	16	$\chi^2(1)$ = 13.375, p=0.000255 ***	**
	P3	20	$\chi^2(1)$ = 5.3366, p=0.02088 *	*
	P4	16	$\chi^2(1)$ = 7.7361, p=0.005413 **	*
	P5	17	$\chi^2(1)$ = 5.3912, p=0.02024 *	*
	P6	18	$\chi^2(1)$ = 6.3356, p=0.01183 *	*
**BASELINE – MEAN PARTICIPATION (CLUSTERED)**	Controls *vs* Patients		$\chi^2(1)$ = 3.4547, p=0.06307 #	
	C1	19		
	C2	19		
	C3	19		
	P1	16	$\chi^2(1)$ = 2.4707, p=0.116	n.s.
	P3	20	$\chi^2(1)$ = 0.0166, p=0.8974	n.s.
	P4	16	$\chi^2(1)$ = 2.361, p=0.1244	n.s.
	P5	17	$\chi^2(1)$ = 8.6113, p=0.003341 **	*
	P6	18	$\chi^2(1)$ = 7.2858, p=0.00695 **	*
**BASELINE – MEAN EVENT AMPLITUDE (CLUSTERED)**	Controls *vs* Patients		$\chi^2(1)$ = 0.5738, p=0.4487	
	C1	19		
	C2	19		
	C3	20		
	P1	16	$\chi^2(1)$ = 1.6833, p=0.1945	n.s.
	P3	20	$\chi^2(1)$ = 0.1984, p=0.656	n.s.
	P4	15	$\chi^2(1)$ = 0.5757, p=0.448	n.s.
	P5	17	$\chi^2(1)$ = 1.2977, p=2546	n.s.
	P6	18	$\chi^2(1)$ = 2.4442, p=0.118	n.s.
**BASELINE - MEAN EVENT AREA (CLUSTERED)**	Controls *vs* Patients		$\chi^2(1)$ = 6.2045, p=0.01274 *	
	C1	19		
	C2	19		
	C3	20		
	P1	16	$\chi^2(1)$ = 1.5116, p=0.2189	n.s.
	P3	20	$\chi^2(1)$ = 1.5038, p=0.2201	n.s.
	P4	15	$\chi^2(1)$ = 1.1258, p=0.2887	n.s.
	P5	16	$\chi^2(1)$ = 3.2189, p=0.07279 #	n.s.
	P6	18	$\chi^2(1)$ = 1.8313, p=0.176	n.s.
**BASELINE – COV MEAN FREQUENCY**	Controls *vs* Patients		$\chi^2(1)$ = 0.2544, p=0.614	
	C1	19		
	C2	19		
	C3	19		
	P1	16	$\chi^2(1)$ = 1.1347, p=0.2868	n.s.
	P3	20	$\chi^2(1)$ = 1.4188, p=0.2336	n.s.
	P4	15	$\chi^2(1)$ = 0.0205, p=0.8862	n.s.
	P5	17	$\chi^2(1)$ = 0.4382, p=0.508	n.s.
	P6	18	$\chi^2(1)$ = 10.009, p=0.001558 **	**
**BASELINE – COV PARTICIPATION**	Controls *vs* Patients		$\chi^2(1)$ = 5.053, p=0.02458 *	
	C1	19		
	C2	18		
	C3	19		
	P1	16	$\chi^2(1)$ = 7.4204, p=0.006449 **	**
	P3	20	$\chi^2(1)$ = 0.26, p=0.6101	n.s.
	P4	15	$\chi^2(1)$ = 9.6444, p=0.001899 **	**
	P5	17	$\chi^2(1)$ = 7.9171, p=0.004897 **	**
	P6	18	$\chi^2(1)$ = 7.9481, p=0.004814 **	**
**BASELINE – FRACTION FULL PARTICIPATION**	Controls *vs* Patients		$\chi^2(1)$ = 4.0279, p=0.04476 *	
	C1	19		
	C2	19		
	C3	20		
	P1	16	$\chi^2(1)$ = 5.0148, p=0.02513 *	#
	P3	20	$\chi^2(1)$ = 0.0051, p=0.9433	n.s.
	P4	15	$\chi^2(1)$ = 1.5839, p=0.2082	n.s.
	P5	17	$\chi^2(1)$ = 7.4712, p=0.006269 **	*
	P6	18	$\chi^2(1)$ = 1.8139, p=0.178	n.s.
**BASELINE – MEAN DURATION (SEC.)**	Controls *vs* Patients		$\chi^2(1)$ = 5.4581, p=0.01948 *	
	C1	19		
	C2	19		
	C3	20		
	P1	16	$\chi^2(1)$ = 2.393, p=0.1219	n.s.

Table S2: Table with statistical parameters

	P3	20	$\chi^2(1) = 0.8486$, p=0.357	n.s.
	P4	16	$\chi^2(1) = 1.1909$, p=0.2752	n.s.
	P5	17	$\chi^2(1) = 2.2839$, p=0.1307	n.s.
	P6	18	$\chi^2(1) = 1.1675$, p=0.2799	n.s.
**BASELINE – MEAN TIME TO PEAK (SEC.)**	Controls *vs* Patients		$\chi^2(1) = 7.0265$, p=0.008031 **	
	C1	19		
	C2	18		
	C3	20		
	P1	16	$\chi^2(1) = 3.2633$, p=0.07085 #	n.s.
	P3	20	$\chi^2(1) = 2.7689$, p=0.09611 #	n.s.
	P4	16	$\chi^2(1) = 1.9223$, p=0.1656	n.s.
	P5	16	$\chi^2(1) = 3.1707$, p=0.07497 #	n.s.
	P6	18	$\chi^2(1) = 0.7459$, p=0.3878	n.s.
**BASELINE – MEAN TIME TO DECAY (SEC.)**	Controls *vs* Patients		$\chi^2(1) = 3.255$, p=0.0712	
	C1	19		
	C2	19		
	C3	20		
	P1	16	$\chi^2(1) = 1.6098$, p=0.2045	n.s.
	P3	20	$\chi^2(1) = 0.046$, p=0.8302	n.s.
	P4	15	$\chi^2(1) = 0.9929$, p=0.319	n.s.
	P5	17	$\chi^2(1) = 1.5913$, p=0.2071	n.s.
	P6	18	$\chi^2(1) = 1.337$, p=0.2476	n.s.
**BASELINE – MEAN INTERBURST INTERVAL (END-START; SEC.)**	Controls *vs* Patients		$\chi^2(1) = 0.8305$, p=0.3621	
	C1	19		
	C2	18		
	C3	20		
	P1	16	$\chi^2(1) = 10.43$, p=0.00124 **	**
	P3	20	$\chi^2(1) = 5.6483$, p=0.01747 *	*
	P4	15	$\chi^2(1) = 7.4298$, p=0.006415 **	**
	P5	17	$\chi^2(1) = 0.1414$, p=0.7069	n.s.
	P6	18	$\chi^2(1) = 5.6919$, p=0.01704 *	*
**BASELINE – COV INTERBURST INTERVAL (END-START; SEC.)**	Controls *vs* Patients		$\chi^2(1) = 7.5934$, p=0.005858 **	
	C1	19		
	C2	19		
	C3	20		
	P1	16	$\chi^2(1) = 2.5452$, p=0.1106	n.s.
	P3	20	$\chi^2(1) = 1.4403$, p=0.2301	n.s.
	P4	14	$\chi^2(1) = 3.1939$, p=0.07391 #	n.s.
	P5	17	$\chi^2(1) = 1.9398$, p=0.1637	n.s.
	P6	18	$\chi^2(1) = 2.6805$, p=0.1016	n.s.
**4AP – FREQUENCY (MIN.)**	Controls *vs* Patients		$\chi^2(1) = 4.0248$, p=0.04484 *	
	C1	18		
	C2	18		
	C3	18		
	P1	15	$\chi^2(1) = 0.8664$, p=0.3519	n.s.
	P3	16	$\chi^2(1) = 0.6956$, p=0.4043	n.s.
	P4	14	$\chi^2(1) = 0.3747$, p=0.5405	n.s.
	P5	17	$\chi^2(1) = 4.9438$, p=0.02618 *	n.s.
	P6	12	$\chi^2(1) = 3.8757$, p=0.04899 *	n.s.
**4AP – MEAN PARTICIPATION (CLUSTERED)**	Controls *vs* Patients		$\chi^2(1) = 13.502$, p=0.0002384 ***	
	C1	18		
	C2	18		
	C3	18		
	P1	15	$\chi^2(1) = 8.7137$, p=0.003158 **	**
	P3	16	$\chi^2(1) = 7.7354$, p=0.005415 **	**
	P4	14	$\chi^2(1) = 8.7055$, p=0.003172 **	**
	P5	17	$\chi^2(1) = 6.7036$, p=0.009622 **	*
	P6	12	$\chi^2(1) = 2.8603$, p=0.09079 #	#
**FOLD CHANGE FREQUENCY (4AP/BASELINE)**	Controls *vs* Patients		$\chi^2(1) = 0.2599$, p=0.6102	
	C1	18		
	C2	18		
	C3	17		
	P1	15	$\chi^2(1) = 3.2992$, p=0.06931 #	n.s.
	P3	15	$\chi^2(1) = 1.2611$, p=0.2615	n.s.
	P4	13	$\chi^2(1) = 1.4548$, p=0.2278	n.s.
	P5	16	$\chi^2(1) = 7.2118$, p=0.007243 **	*
	P6	11	$\chi^2(1) = 7.8259$, p=0.00515 **	*
**4AP – MEAN EVENT AMPLITUDE (CLUSTERED)**	Controls *vs* Patients		$\chi^2(1) = 0.7538$, p=0.3853	
	C1	18		
	C2	18		
	C3	18		
	P1	15	$\chi^2(1) = 0.0494$, p=0.8241	n.s.
	P3	16	$\chi^2(1) = 0.033$, p=0.8558	n.s.

Table S2: Table with statistical parameters

	P4	14	$\chi^2(1) = 0.4607$, p=0.4973	n.s.
	P5	15	$\chi^2(1) = 0.8751$, p=0.3495	n.s.
	P6	12	$\chi^2(1) = 0.9758$, p=0.3232	n.s.
**4AP – MEAN EVENT AREA (CLUSTERED)**	Controls *vs* Patients		$\chi^2(1) = 4.7342$, p=0.02957 *	
	C1	18		
	C2	18		
	C3	18		
	P1	15	$\chi^2(1) = 1.2263$, p=0.2681	n.s.
	P3	16	$\chi^2(1) = 0.4801$, p=0.4884	n.s.
	P4	14	$\chi^2(1) = 1.4046$, p=0.2359	n.s.
	P5	14	$\chi^2(1) = 3.4259$, p=0.06418 #	n.s.
	P6	12	$\chi^2(1) = 1.5256$, p=0.2168	n.s.
**4AP – COV FREQUENCY**	Controls *vs* Patients		$\chi^2(1) = 5.1854$, p=0.02278 *	
	C1	18		
	C2	17		
	C3	18		
	P1	15	$\chi^2(1) = 11.674$, p=0.0006338 ***	*
	P3	16	$\chi^2(1) = 6.5335$, p=0.01059 *	**
	P4	14	$\chi^2(1) = 15.381$, p=0.00008787 ***	***
	P5	15	$\chi^2(1) = 4.0619$, p=0.04386 *	*
	P6	12	$\chi^2(1) = 6.5406$, p=0.01054 *	*
**4AP – COV PARTICIPATION**	Controls *vs* Patients		$\chi^2(1) = 7.9396$, p=0.004836 **	
	C1	18		
	C2	18		
	C3	17		
	P1	15	$\chi^2(1) = 10.166$, p=0.00143 **	**
	P3	16	$\chi^2(1) = 6.7254$, p=0.009505 **	*
	P4	14	$\chi^2(1) = 11.421$, p=0.0007263 ***	**
	P5	15	$\chi^2(1) = 4.5013$, p=0.03387 *	*
	P6	12	$\chi^2(1) = 2.9367$, p=0.08659 #	#
**4AP – FRACTION FULL PARTICIPATION**	Controls *vs* Patients		$\chi^2(1) = 15.195$, p=0.00009696 ***	
	C1	17		
	C2	18		
	C3	18		
	P1	15	$\chi^2(1) = 5.8857$, p=0.01526 *	*
	P3	16	$\chi^2(1) = 5.0075$, p=0.02524 *	*
	P4	14	$\chi^2(1) = 5.763$, p=0.01637 *	*
	P5	15	$\chi^2(1) = 6.5201$, p=0.01067 *	*
	P6	12	$\chi^2(1) = 4.4369$, p=0.03517 *	*
**4AP – MEAN DURATION (SEC.)**	Controls *vs* Patients		$\chi^2(1) = 4.2083$, p=0.04023 *	
	C1	18		
	C2	18		
	C3	18		
	P1	15	$\chi^2(1) = 1.0835$, p=0.2979	n.s.
	P3	16	$\chi^2(1) = 0.3593$, p=0.5489	n.s.
	P4	15	$\chi^2(1) = 0.9108$, p=0.3399	n.s.
	P5	15	$\chi^2(1) = 2.8879$, p=0.08925 #	n.s.
	P6	12	$\chi^2(1) = 1.6233$, p=0.2026	n.s.
**4AP – MEAN TIME TO PEAK (SEC.)**	Controls *vs* Patients		$\chi^2(1) = 5.5883$, p=0.01808 *	
	C1	18		
	C2	18		
	C3	18		
	P1	15	$\chi^2(1) = 0.8271$, p=0.3631	n.s.
	P3	15	$\chi^2(1) = 1.4508$, p=0.2284	n.s.
	P4	15	$\chi^2(1) = 1.4967$, p=0.2212	n.s.
	P5	15	$\chi^2(1) = 2.4054$, p=0.1209	n.s.
	P6	12	$\chi^2(1) = 1.4068$, p=0.2356	n.s.
**4AP – MEAN TIME TO DECAY (SEC.)**	Controls *vs* Patients		$\chi^2(1) = 3.8656$, p=0.04929 *	
	C1	18		
	C2	18		
	C3	18		
	P1	15	$\chi^2(1) = 1.8612$, p=0.1725	n.s.
	P3	16	$\chi^2(1) = 0.1184$, p=0.7308	n.s.
	P4	14	$\chi^2(1) = 0.702$, p=0.4021	n.s.
	P5	15	$\chi^2(1) = 3.3304$, p=0.06801 #	n.s.
	P6	12	$\chi^2(1) = 1.8002$, p=0.1797	n.s.
**4AP – MEAN INTERBURST INTERVAL (END-START: SEC.)**	Controls *vs* Patients		$\chi^2(1) = 5.2988$, p=0.02134 *	
	C1	18		
	C2	17		
	C3	18		
	P1	14	$\chi^2(1) = 2.8293$, p=0.09256 #	n.s.
	P3	15	$\chi^2(1) = 1.2212$, p=0.2691	n.s.
	P4	14	$\chi^2(1) = 0.0778$, p=0.7803	n.s.
	P5	15	$\chi^2(1) = 7.8658$, p=0.005038 **	*
	P6	11	$\chi^2(1) = 7.2182$, p=0.007217 **	*
**4AP – COV INTERBURST INTERVAL (END-START: SEC.)**	Controls *vs* Patients		$\chi^2(1) = 8.8725$, p=0.002895 **	
	C1	18		

Table S2: Table with statistical parameters

	C2	18		
	C3	18		
	P1	15	$\chi^2(1) = 3.2569$, p=0.07112 #	#
	P3	16	$\chi^2(1) = 4.3693$, p=0.03659 *	#
	P4	14	$\chi^2(1) = 4.514$, p=0.03362 *	#
	P5	15	$\chi^2(1) = 5.1212$, p=0.02363 *	#
	P6	12	$\chi^2(1) = 1.097$, p=0.2949	n.s.

LDA ANALYSIS	
**EPHYS LDA-CONDITION**	n.s.
**EPHYS LDA-LINE**	n.s.
**CALCIUM IMAGING LDA-CONDITION**	$p < 10^{-4}$
**CALCIUM IMAGING LDA-LINE**	$p < 10^{-4}$

P values according to lookup table Combrisson et al., 2015.

Table S3: Table with qPCR primers

GAPDH -FW	GTTCGACAGTCAGCCGCATC
GAPDH - RV	TCCGTTGACTCCGACCTTCA
MYC -FW	CCCTCCACTCGGAAGGACTA
MYC -RV	GCTGGTGCATTTTCGGTTGT
STXBP1 Center-FW	AGGTGTCCCAGGAAGTC
STXBP1 Center -RV	CTGAGCTCTTTCTGGTACTG
STXBP1 5’-FW	TCCAGATGCCCTGTTTAATG
STXBP1 5’ -RV	CCAAGGAATAGACCTGGGAT
STXBP1 3’-FW	GTCACCGATTCCACGCT
STXBP1 3’ -RV	TCCTTGATAATCGGAGTCCA
Snap25 -FW	TTCATCCGCAGGGTAACA
Snap25 RV	ATCGATCTCATTGCCCATATC
Vamp1 -FW	CTCTGTCAGAAAAATGTCTGCT
Vamp1 -RV	GTTTGCTGTAGTCGTCTGTT
Stx1A -FW	GTTCCAAGTTAAAGAGCATCG
Stx1A -RV	TTGTACTCCGACATGACCTC
Stx1B -FW	CCAAATTGAAAGCGATCGAG
Stx1B -RV	TCGCGTTATATTCGGTCATT

FW = Forward; RV = Reverse

Table S5: Table with detailed information on control iPSC lines

Line	Name	Age of biopsy	Sex	Source	Characterized in
Control 1	GM25256	30 yo (Asian)	m	Coriell	Kreitzer et al. (2013)
Control 2	GM23973	19 yo (Caucasian/European)	m	Coriell	Holmes et al., (2017)
Control 3	hVS-88	Anonymous, 74 days old infant	m	Collaborator	Nadadhur et al., (2019)
